# Structure of *Mycobacterium tuberculosis* cytochrome *bcc* in complex with Q203 and TB47, two anti-TB drug candidates

**DOI:** 10.7554/eLife.69418

**Published:** 2021-11-25

**Authors:** Shan Zhou, Weiwei Wang, Xiaoting Zhou, Yuying Zhang, Yuezheng Lai, Yanting Tang, Jinxu Xu, Dongmei Li, Jianping Lin, Xiaolin Yang, Ting Ran, Hongming Chen, Luke W Guddat, Quan Wang, Yan Gao, Zihe Rao, Hongri Gong

**Affiliations:** 1 State Key Laboratory of Medicinal Chemical Biology, College of Pharmacy, Nankai University Tianjin China; 2 State Key Laboratory of Medicinal Chemical Biology, College of Life Sciences, Nankai University Tianjin China; 3 Shanghai Institute for Advanced Immunochemical Studies and School of Life Science and Technology, ShanghaiTech University Shanghai China; 4 Bioland Laboratory (Guangzhou Regenerative Medicine and Health - Guangdong Laboratory) Guangzhou China; 5 School of Chemistry and Molecular Biosciences, The University of Queensland Brisbane Australia; 6 National Laboratory of Biomacromolecules, CAS Center for Excellence in Biomacromolecules, Institute of Biophysics Beijing China; 7 Laboratory of Structural Biology, Tsinghua University Beijing China; Stockholm University Sweden; Goethe University Germany

**Keywords:** Mycobacterium tuberculosis, cytochrome bcc complex, cryo-electron microscopy, Q203, TB47, Other

## Abstract

Pathogenic mycobacteria pose a sustained threat to global human health. Recently, cytochrome *bcc* complexes have gained interest as targets for antibiotic drug development. However, there is currently no structural information for the cytochrome *bcc* complex from these pathogenic mycobacteria. Here, we report the structures of *Mycobacterium tuberculosis* cytochrome *bcc* alone (2.68 Å resolution) and in complex with clinical drug candidates Q203 (2.67 Å resolution) and TB47 (2.93 Å resolution) determined by single-particle cryo-electron microscopy. *M. tuberculosis* cytochrome *bcc* forms a dimeric assembly with endogenous menaquinone/menaquinol bound at the quinone/quinol-binding pockets. We observe Q203 and TB47 bound at the quinol-binding site and stabilized by hydrogen bonds with the side chains of _QcrB_Thr^313^ and _QcrB_Glu^314^, residues that are conserved across pathogenic mycobacteria. These high-resolution images provide a basis for the design of new mycobacterial cytochrome *bcc* inhibitors that could be developed into broad-spectrum drugs to treat mycobacterial infections.

## Introduction

**Figure 1.**
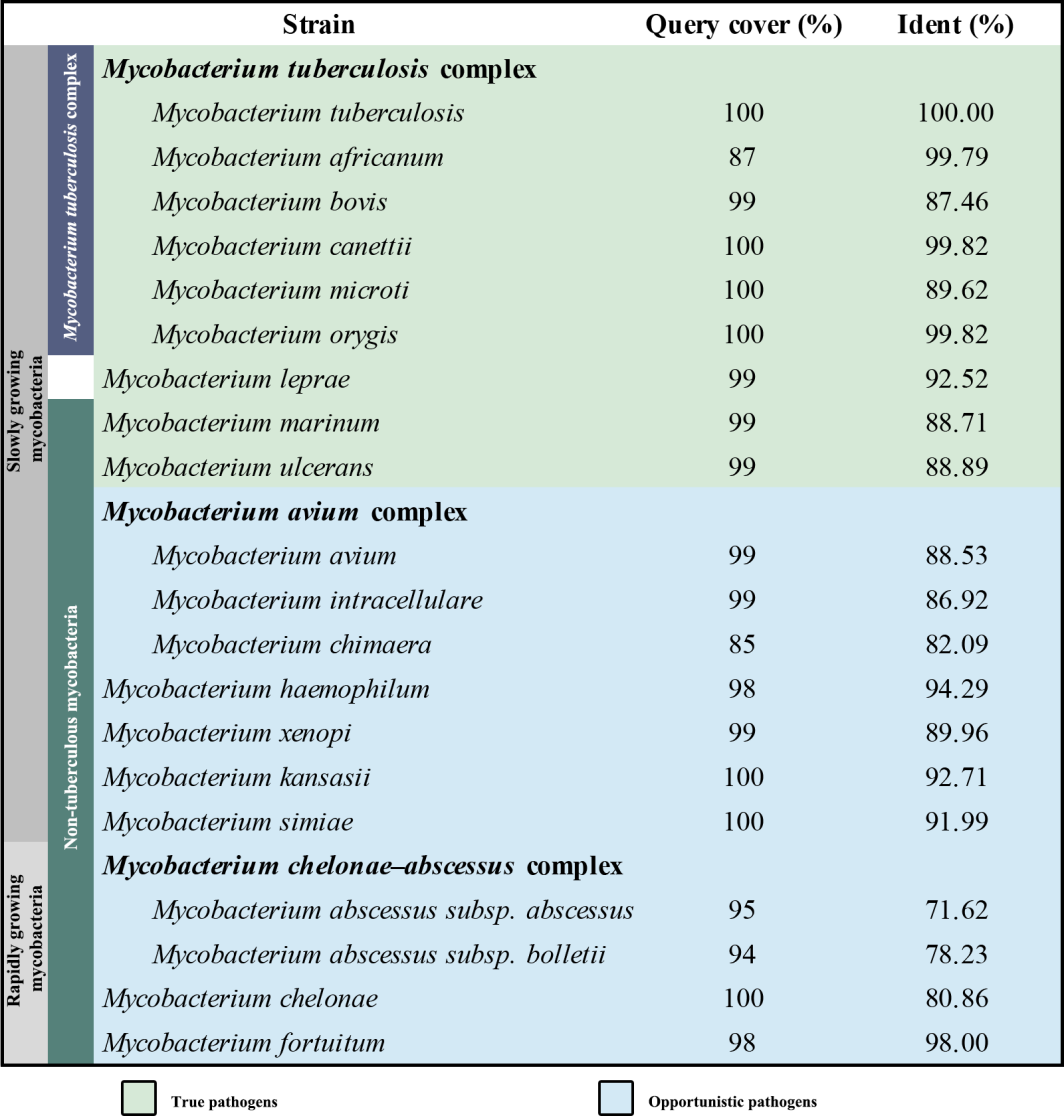
Sequence similarity comparison of *M*. *tuberculosis* QcrB with other pathogenic mycobacteria.

Mycobacteria, which belong to the phylum Actinobacteria, have coevolved with humans over thousands of years (Chisholm et al., 2016). Approximately 200 species of mycobacteria have been identified that have diverse lifestyles, morphologies, and metabolic pathways (Tortoli et al., 2017). Mycobacteria can broadly be grouped into two categories: tuberculosis-causing mycobacteria and non-tuberculous mycobacteria (NTM). *Mycobacterium leprae* is often represented in a distinct genetic clade owing to its genetic and phenotypic differences compared to other mycobacterial species (Cole et al., 2001). Mycobacteria can be further classified into fast-growing and slow-growing species or species complexes; these assignments are according to the physiological, phenotypic and phylogenetic characteristics (Rastogi et al., 2001). Although nearly all mycobacteria are saprophytes or non-pathogenic to humans, a few species cause diseases, resulting in pulmonary and extra-pulmonary infections that can affect nearly all organs. Infections, which are caused by strict or opportunistic pathogenic mycobacteria (Figure 1), pose a sustained threat to human health. Of these, tuberculosis (TB), caused by *Mycobacterium tuberculosis* (*M. tuberculosis*), is the most serious, leading to ~1.2 million fatalities per year (World Health Organization, 2019). Infections involving other pathogenic mycobacteria, for example, *Mycobacterium abscessus* and *Mycobacterium avium complex*, are on the rise with some outnumbering those caused by *M. tuberculosis* in countries including the United States (Donohue, 2018; Johansen et al., 2020). These infections are notoriously difficult to treat due to intrinsic or emerging resistance to many common antibiotics, thus exacerbating the challenge to find suitable drug targets.

Oxidative phosphorylation (OXPHOS) has gained interest as a target space for antibiotic drug development (Cook et al., 2017; Hards et al., 2020). In OXPHOS, the protein complexes of the electron transport chain (ETC) establish a proton motive force (PMF) across a biomembrane that drives the synthesis of adenosine triphosphate (ATP) by ATP synthase (Mitchell, 1961). Maintenance of PMF and ATP homeostasis is required for the survival of both replicative and non-replicative (often referred to as dormant) mycobacteria, and its dissipation leads to a rapid loss of cell viability and cell death (Koul et al., 2008; Rao et al., 2008). The reliance on the PMF and ATP homeostasis thus highlights the importance of the mycobacterial proton-pumping cytochrome *bcc-aa*_3_ supercomplex, which consists of a *bcc* menaquinol reductase (complex III, CIII) and an *aa*_3_ oxidase (complex IV, CIV) that are tightly associated (Gong et al., 2018; Kim et al., 2015; Megehee et al., 2006). Several studies support the attractiveness of cytochrome *bcc-aa*_3_ for mycobacterial drug development (de Jager et al., 2020; Liu et al., 2019; Lu et al., 2019; Pethe et al., 2013; Scherr et al., 2018). Given the strict sequence conservation of this complex (Figure 1), broad-spectrum activity of a therapeutic within the pathogenic mycobacteria is likely (Lee et al., 2020b). Interestingly, all the cytochrome *bcc-aa*_3_ inhibitors published to date appear to target the QcrB subunit (Figure 1) of the cytochrome *bcc* complex and are likely bound to the menaquinol-binding (Qp) site of the QcrB subunit (Lee et al., 2020b). The most advanced of these are Q203 and TB47, which have been shown to clear infections due to *M. tuberculosis* (de Jager et al., 2020; Lu et al., 2019; Pethe et al., 2013) and *Mycobacterium ulcerans* (Liu et al., 2019; Scherr et al., 2018). Q203 has recently completed phase II clinical trials for TB treatment (ClinicalTrials.gov number, NCT03563599; de Jager et al., 2020). TB47 has also been evaluated in a preclinical study (http://www.newtbdrugs.org/pipeline/clinical).

To progress an understanding of cytochrome *bcc* and its interaction with new drug leads, here we have determined the atomic resolution cryo-electron microscopy (cryo-EM) structures of *M. tuberculosis* cytochrome *bcc* alone (2.68 Å resolution) and in complex with Q203 (2.67 Å resolution) and TB47 (2.93 Å resolution). These high-resolution structures will greatly accelerate efforts towards structure-guided drug discovery.

## Results and discussion

### Structure of *M. tuberculosis* cytochrome *bcc*

**Figure 2.**
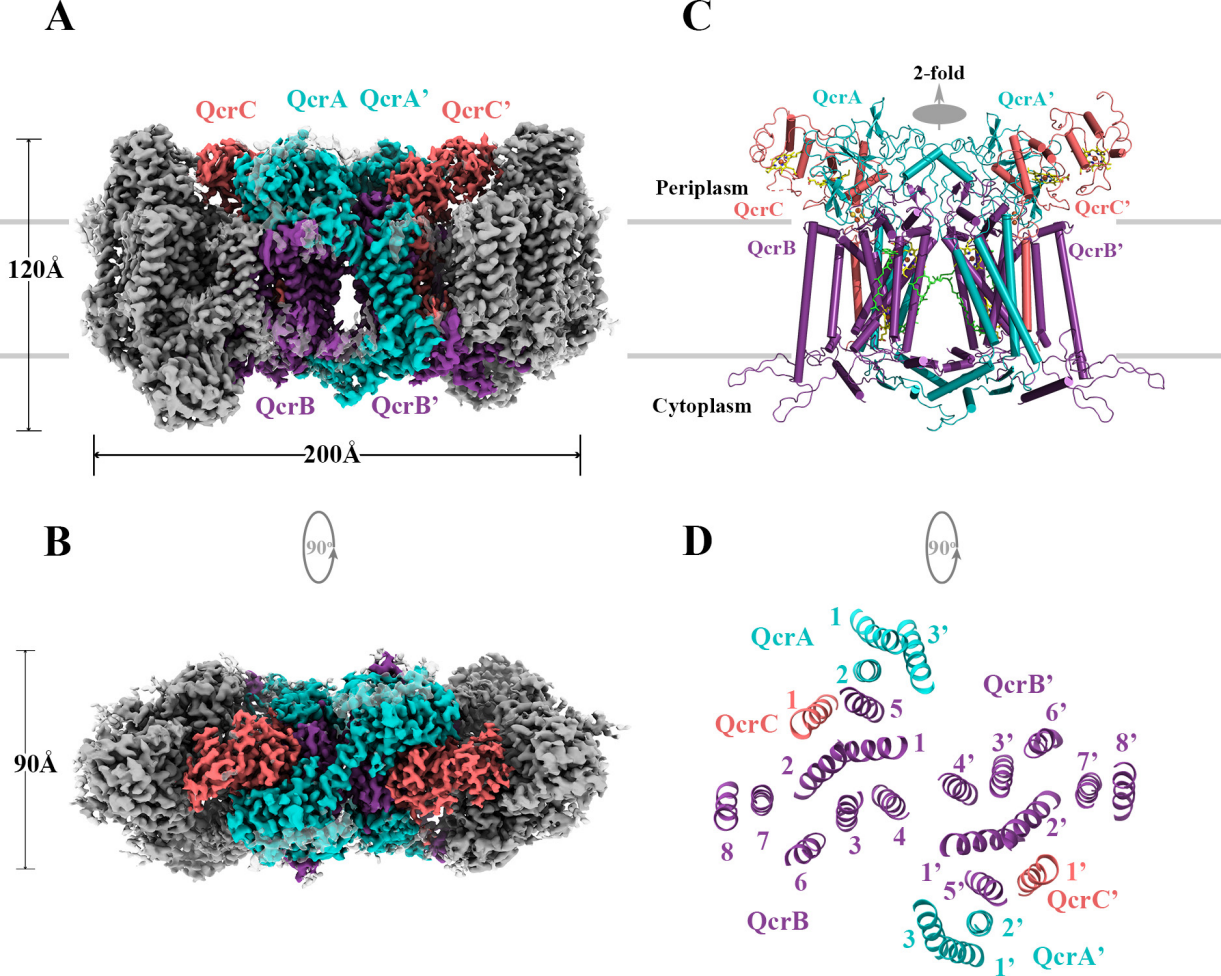
Overall architecture of the hybrid supercomplex. (**A**) Front view and (**B**) top view of the cryo-electron microscopy (cryo-EM) map of hybrid supercomplex at 2.68 Å resolution. QcrA, QcrB, and QcrC of the *M. tuberculosis* cytochrome *bcc* are colored teal, purple, and salmon, respectively. Other subunits of the hybrid supercomplex are in gray. (**C**) Cartoon representation of cytochrome *bcc*, using the same color scheme as above. The twofold symmetry of the dimer is depicted by the gray axis. The heme groups (*b*_H_, *b*_L_, *c*_D1_, and *c*_D2_) and menaquinone/menaquinol (MK_P_/MK_N_) are shown as stick models. The [2Fe-2S] clusters are shown as spheres. (**D**) A cross-sectional view (top) of the helices in the cytochrome *bcc* dimer. Figure 2—source data 1.Oxygen consumption of the hybrid supercomplex measures using Clark-type oxygen electrode.

**Figure 3.**
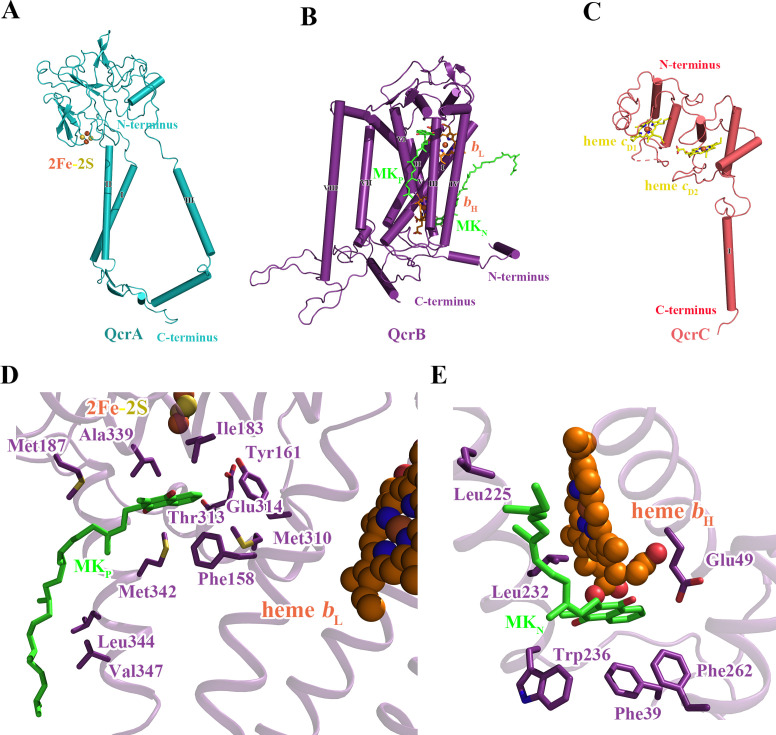
Structure of the *M. tuberculosis* cytochrome *bcc* subunits. Cartoon representation of the monomers of (**A**) QcrA, (**B**) QcrB, and (**C**) QcrC, with prosthetic groups. (**D**) The Q_P_-binding site and (**E**) Q_N_-binding site. The residues potentially involved in the binding of MK/MKH_2_ are shown with side chains in stick model representation. MK/MKH_2_ have their carbon atoms in green and are represented as stick models. The [2Fe-2S] and heme groups are shown as spheres and labeled.

Considering that the hybrid supercomplex consisting of *M. tuberculosis* CIII and *Mycobacterium smegmatis* CIV can be stabilized as a functional assembly (Kim et al., 2015), we first chose to express and purify this complex to a high level of homogeneity (Figure 2—figure supplement 1). We confirmed this complex to be active with a turnover number of 23.3 ± 2.4 e^-^s^–1^ (mean ± SD, n = 4), in agreement with the previous study that shows *M. tuberculosis* CIII can functionally complement native *M. smegmatis* CIII and maintain the growth of *M. smegmatis* (Kim et al., 2015). The structure of this hybrid supercomplex was determined by cryo-EM to an overall resolution of 2.68 Å, allowing that the components to be clearly assigned in the density map (Figure 2—figure supplements 2 and 3, Table 1). The hybrid supercomplex including the *M. tuberculosis* CIII dimer forms a pseudo twofold symmetrical compact rod, but with a slight curvature in the membrane plane, as previously observed in the *M. smegmatis* CIII_2_CIV_2_ supercomplex (Gong et al., 2018; Wiseman et al., 2018; Figure 2A and B). The hybrid supercomplex is structurally similar to the supercomplex isolated from *M. smegmatis* (Gong et al., 2018; Wiseman et al., 2018; Figure 2—figure supplement 4), except that subunits LpqE and Unk (probably MSMEG_0987) (Wiseman et al., 2018) were not observed here. The absence of these two subunits may be due to their depletion during purification of the supercomplex or their map density signal was averaged to background noise during structural determination. As expected, the topology of *M. tuberculosis* cytochrome *bcc* and *M. smegmatis* CIV in the hybrid supercomplex is also similar to that of the equivalent subunits in the *M. smegmatis* CIII_2_CIV_2_ supercomplex (Figure 2C and D, Figure 2—figure supplements 4 and 5). As such, there is no notable non-native contacts that resulted from the hybrid assembly in the hybrid supercomplex. This is attributable to the high structural similarity between the *M. tuberculosis* cytochrome *bcc* and *M. smegmatis* cytochrome *bcc*. In *M. tuberculosis* cytochrome *bcc*, QcrA has three transmembrane helices (TMHs) and has a ‘U’-shaped structure (Figures 2D and 3A). The N-terminal region with TMH1/2 and the TMH3 make up the two arms of the ‘U’ structure. These arms are linked by the region located near the cytoplasmic side. Attached to _QcrA_TMH3 is the C-terminal domain, which faces the periplasmic side of the membrane and holds the [2Fe-2S] cluster in place. QcrB has eight TMHs (Figures 2D and 3B). Four of these are responsible for burying two functionally important heme *b* cofactors (high potential heme *b*_H_ and low potential heme *b*_L_). Heme *b*_L_ and heme *b*_H_ are located between TMH I/II and TMH III/IV, respectively. Heme *b*_L_ is near the periplasmic side and heme *b*_H_ near the cytoplasmic side. QcrC is a transmembrane protein with a C-terminal TMH located between _QcrB_TMH5 and _QcrB_TMH7 (Figures 2D and 3C). The N-terminal periplasmic portion of QcrC can be divided into two heme-containing cytochrome *c* domains designated D1 and D2. The D1 domain protrudes out of the core of CIII, whereas the D2 domain interacts extensively with QcrA and QcrB. In the *M. smegmatis* CIII_2_CIV_2_ supercomplex, the cytochrome *cc* head domain of QcrC adopts an ‘open’ or a ‘closed’ conformation (Gong et al., 2018; Wiseman et al., 2018). However, in this structure it is only the closed conformation (Figure 2—figure supplement 4). Considering the high topology similarity of the subunits and the arrangement of prosthetic groups compared to previous *M. smegmatis* CIII_2_CIV_2_ supercomplex, the mechanism of action of the hybrid supercomplex including *M. tuberculosis* cytochrome *bcc* is expected to be the same as that for the *M. smegmatis* CIII_2_CIV_2_ supercomplex (Gong et al., 2018).

**Figure 2—figure supplement 1.**
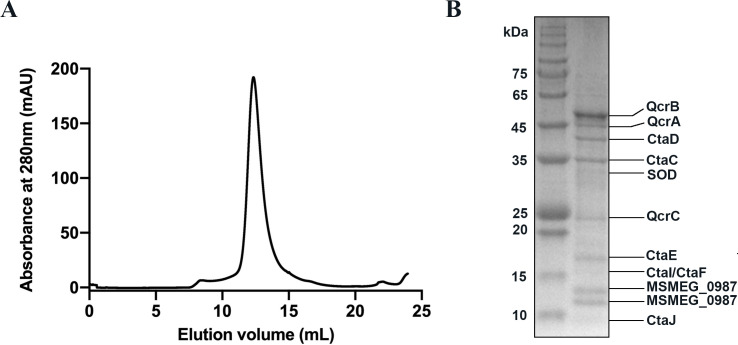
Purification and identification of the hybrid supercomplex consisting of *M. tuberculosis* CIII and *M. smegmatis* CIV. (**A**) The elution profile of the hybrid supercomplex from size-exclusion chromatography (SEC). (**B**) SDS-PAGE of the pooled fraction from the SEC in (**A**). Figure 2—figure supplement 1—source data 1.The elution profile of the hybrid supercomplex from size-exclusion chromatography. Figure 2—figure supplement 1—source data 2.SDS-PAGE of the pooled fraction from the size-exclusion chromatography.

**Figure 2—figure supplement 2.**
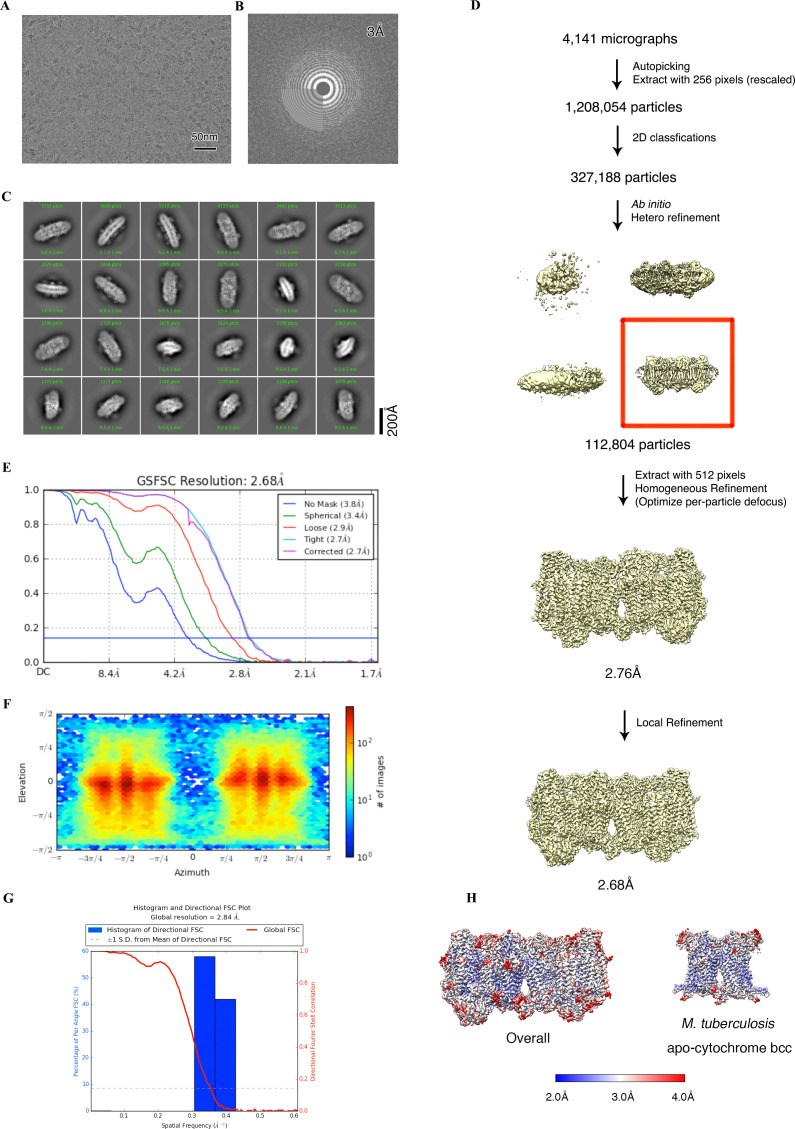
Cryo-electron microscopy (cryo-EM) data processing of the apo hybrid supercomplex consisting of *M. tuberculosis* CIII and *M. smegmatis* CIV. (**A**) Representative electron micrograph of the cryo-EM sample. (**B**) CTF fit of the motion-corrected micrographs. (**C**) Representative 2D classification averages calculated from selected particles. (**D**) Workflow of data processing for the apo hybrid supercomplex. (**E**) Fourier shell correlation (FSC) curves of 3D reconstructions. (**F**) View direction of all particles used in the final 3D reconstruction. (**G**) 3D FSC histogram of the final map. (**H**) The overall and *Mtb* cytochrome *bcc* maps, colored according to the local resolution.

**Figure 2—figure supplement 3.**
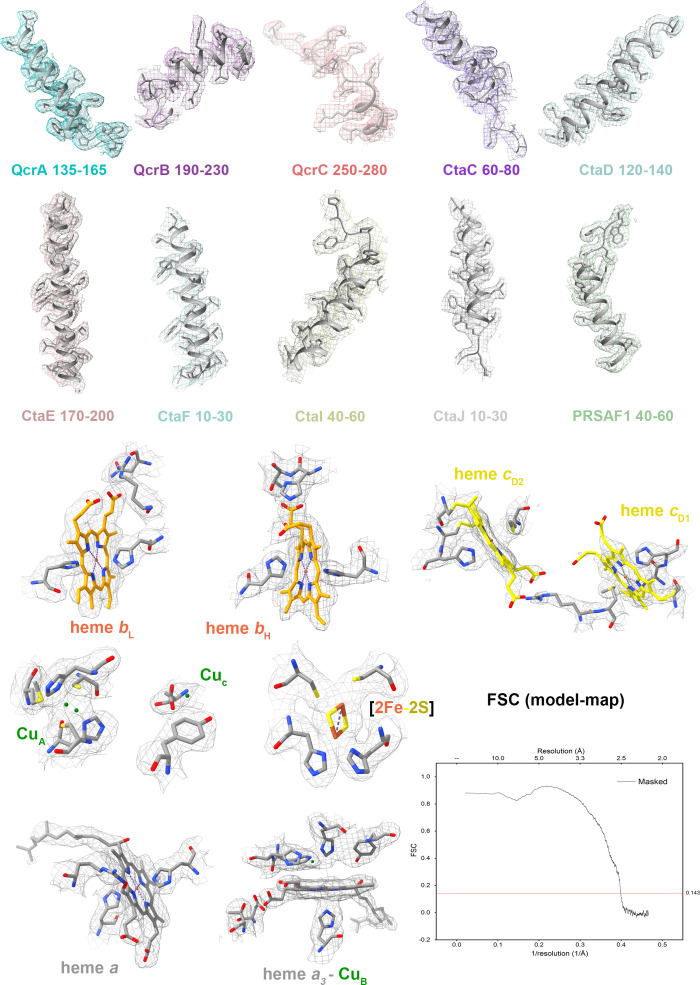
Cryo-electron microscopy (cryo-EM) map quality assessment of the hybrid supercomplex. Representative cryo-EM densities of regions within individual subunits and prosthetic groups. Corresponding subunits with residues and prosthetic groups shown in cartoon representation or as stick models. Map-model Fourier shell correlation (FSC).

**Figure 2—figure supplement 4.**
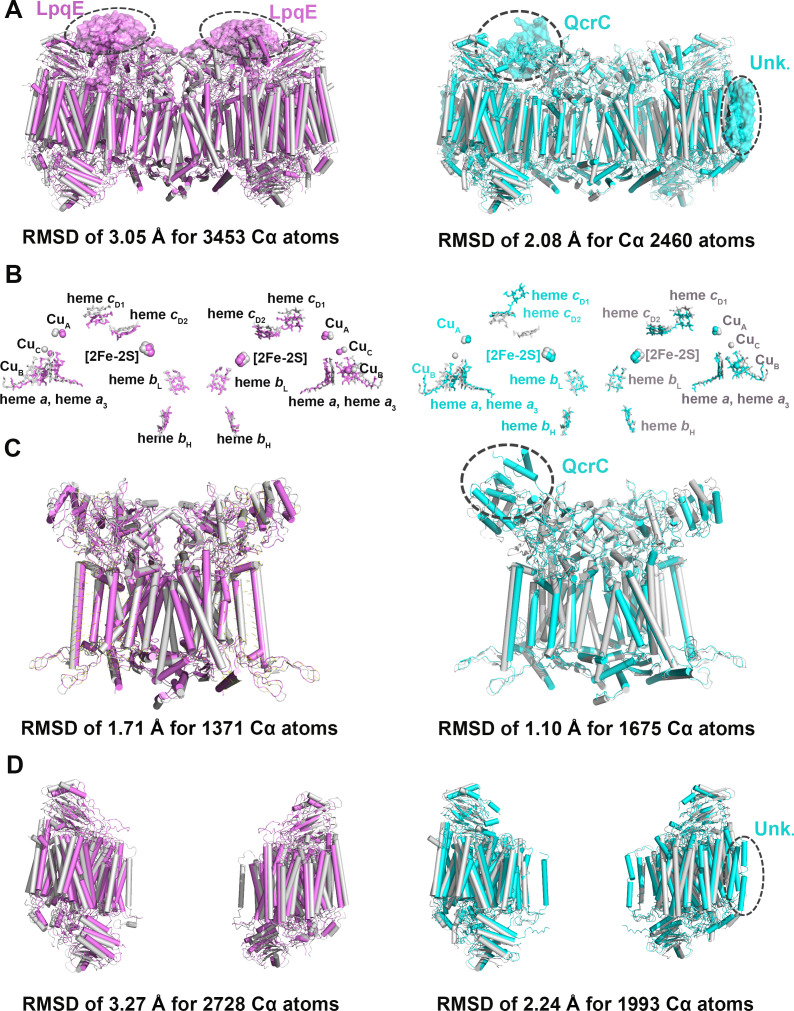
Structural comparison of the hybrid supercomplex III_2_IV_2_ and native CIII_2_CIV_2_ supercomplexes from *M. smegmatis*. (**A**) Superimposition of the hybrid supercomplex III_2_IV_2_ (gray) and *M. smegmatis* CIII_2_CIV_2_ supercomplex shown in magenta (PDB 6ADQ) and cyan (PDB 6HWH), respectively. (**B**) Comparison of prosthetic groups after superimposition. (**C**) Comparison of CIII_2_, using the same color scheme as above. (**D**) Comparison of CIV, using the same color scheme as above.

**Figure 2—figure supplement 5.**
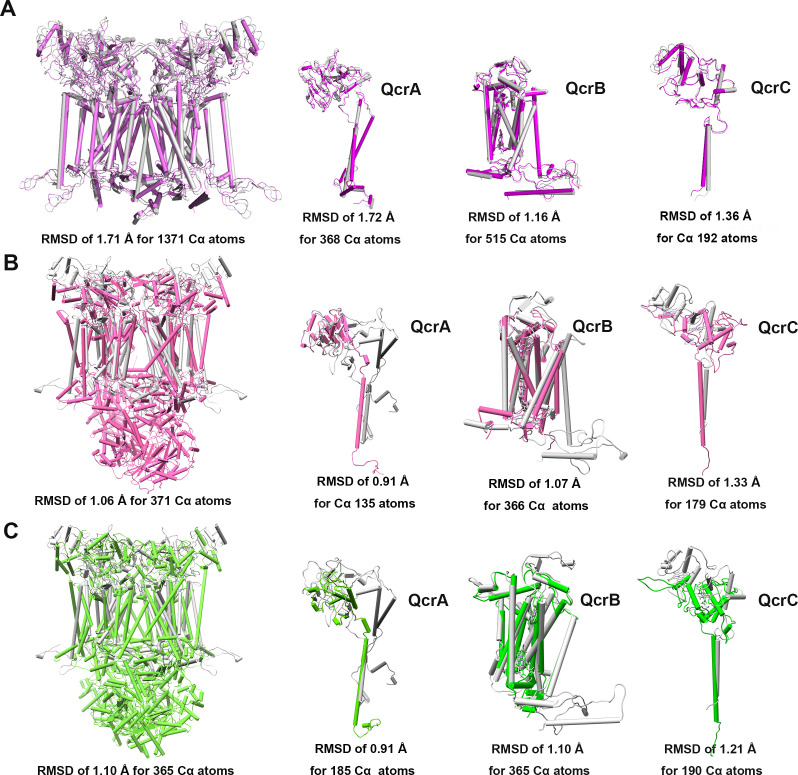
Structural alignment between *M. tuberculosis* CIII and equivalent CIIIs from other species. These complexes are from *M. tuberculosis* (gray), *M. smegmatis* (violet, PDB: 6ADQ) (**A**), *S. cerevisiae* (pink, PDB: 1KYO) (**B**), and *Homo sapiens* (green; PDB: 5XTE) (**C**).

**Figure 3—figure supplement 1.**
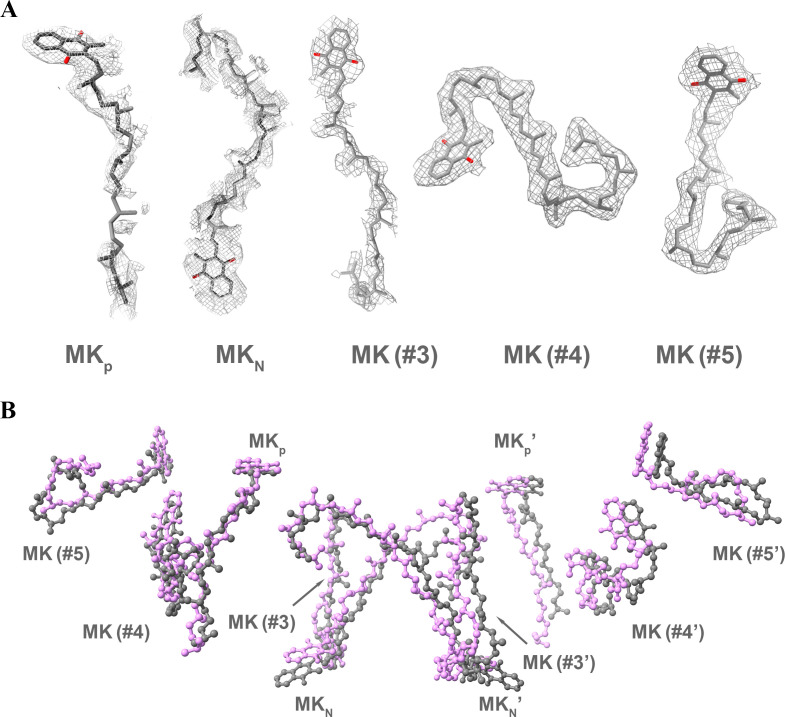
Identification and comparison of MK/MKH_2_ in the hybrid supercomplex. Ten MK/MKH_2_ (gray) have been assigned according to the densities (**A**) and are in the same locations as observed in the *M. smegmatis* CIII_2_CIV_2_ supercomplex (magenta) (PDB 6ADQ) (**B**).

**Figure 3—figure supplement 2.**
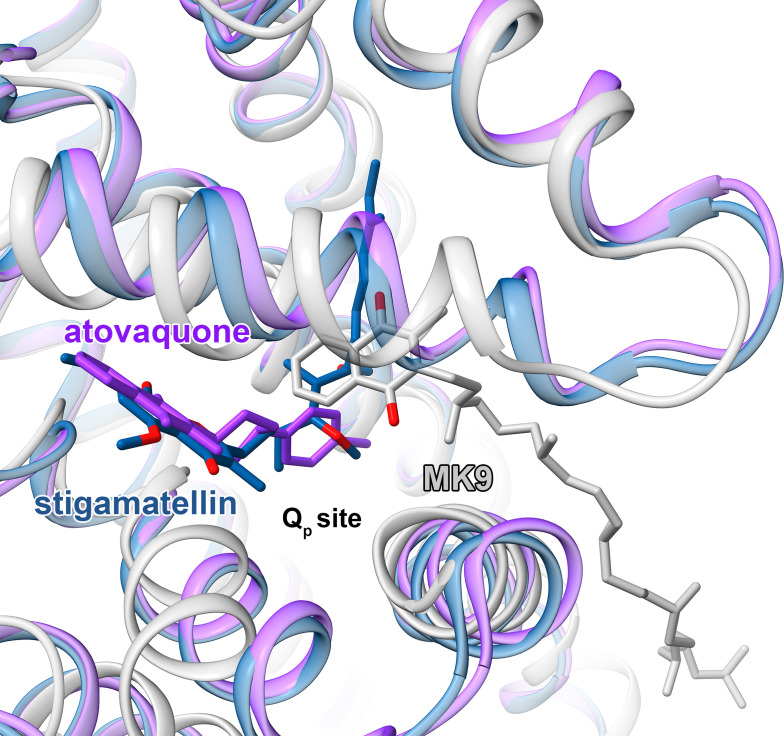
Comparison of *M*. *tuberculosis* CIII quinol-binding site (gray) with those from CIII from *S. cerevisiae* (PDB 3CX5) (blue) and (PDB 4PD4) (purple).

**Figure 3—figure supplement 3.**
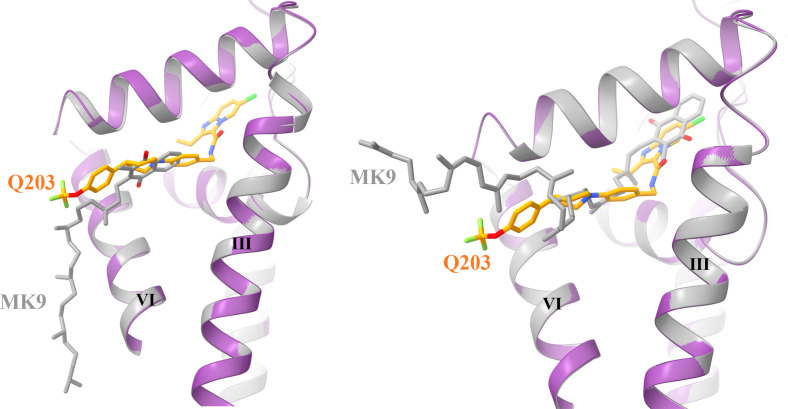
Structural superposition of native (left) and docked (right) MK9 with the Q203 in the Qp site. The QcrB is extracted from the hybrid supercomplex with Q203 (purple) and MK9 (gray) bound is shown in cartoon representation.

**Table 1.** Cryo-electron microscopy data collection, refinement, and validation statistics of hybrid supercomplex.

**State**	**apo**
*Data collection*	
Microscope	Titan Krios
Voltage (kV)	300
Magnification	29,000×
Detector	Gatan K3
Data collection software	SerialEM
Electron exposure (e^–^/Å^2^)	60
Defocus range (μm)	–1.2 to –1.8
Pixel size (Å)	0.82
*Data processing*	
Number of micrographs	4141
Final particle images	112,804
Symmetry imposed	C1
*Map resolution (Å)*Fourier shell correlation 0.143 threshold	
	2.68
*Refinement*	
Initial model used (PDB code)	6ADQ
Map sharpening B factor (Å^2^)d FSC model (0.143) masked	–65.32.5
Map correlation coefficient	0.89
Mean CC for ligands	0.78
*Model composition*	
Non-hydrogen atoms	42,279
Protein residues	5122
*Ligands*	
	9Y0: 2
	CDL: 17
	9YF: 4
	HEA: 4
	HEC: 4
	MQ9: 10
	HEM: 4
	PLM: 4
	CU: 8
	FES: 2

*Root mean squared deviations*	
Bond lengths (Å)	0.005
Bond angles (°)	1.057
*Validation*	
MolProbity score	1.86
Clashscore	7.26
Poor rotamers (%)	0.05
*Ramachandran plot*	
Favored (%)	92.76
Allowed (%)	6.97
Outliers (%)	0.28
Cβ outliers (%)	0.00

### Quinone and quinone-binding pockets of *M. tuberculosis* cytochrome *bcc*

Quinone-binding sites are essential to the function of respiratory chain complexes and thus are good targets for drug discovery (Harikishore et al., 2021; Lee et al., 2020b). Structurally diverse quinones such as ubiquinone and menaquinone (MK) bind in the mitochondrial ETC (Zhang et al., 1998) and mycobacterial ETC (Gong et al., 2018; Wiseman et al., 2018), respectively, indicating that there are structural differences in the quinone-binding sites of the different species. Structural differences in the ubiquinone-binding site are also observed when the mitochondrial and *Escherichia coli* respiratory chain complex IIs are compared (Huang et al., 2021). Therefore, it is feasible that species-specific quinone-binding-site inhibitors could be designed. In the present structure, two canonical quinone-binding sites are identified (Figure 3D and E): (i) the quinol oxidation site (Q_P_ site) and (ii) the quinone reduction site (Q_N_ site). In addition, other quinone-binding sites with quinone bound are also identified as observed in our previous study (Gong et al., 2018; Figure 3—figure supplement 1). These quinones are remote from the canonical quinone-binding sites and into the membrane space, suggesting that they have a structural rather than functional role. The Q_P_ site responsible for menaquinol (MKH_2_) oxidation is near heme *b*_L_, whereas the Q_N_ site responsible for MK reduction is close to heme *b*_H_ (Figure 3D and E, Figure 3—figure supplement 1). The Q_P_ site is at the center of an inverted triangle structure surrounded by helices (Figure 3D). One MK molecule was identified at this site with its naphthoquinone ring surrounded by hydrophobic residues, _QcrB_Phe^158^, _QcrB_Tyr^161^, _QcrB_Leu^180^, _QcrB_Ile^183^, _QcrB_Met^310^, and _QcrB_Met^342^. Its hydrophobic tail, which contains multiple isoprenoid groups, wraps around _QcrB_TMH6 down to its cytoplasmic end and in so doing interacts with _QcrB_Met^187^, _QcrB_Ala^339^, _QcrB_Leu^344^, and _QcrB_Val^347^. The edge-to-edge distance from MK to heme *b*_L_ is 15 Å, exceeding the 14 Å limit for efficient physiological electron transfer (Page et al., 1999). Furthermore, there are no observed hydrogen bonds to the carbonyl groups of MK, which are needed to help deprotonate MKH_2_. In addition, structural superposition with the inhibitor-bound *bc*_1_ complex also shows that the MK binds deeper into the Q_P_ pocket in the *bc*_1_ complex (Birth et al., 2014; Solmaz and Hunte, 2008) than in the currently described complex (Figure 3—figure supplement 2). Hence, the endogenous electron donor MKH_2_ should bind deeper inside the pocket and close to polar residues such as _QcrB_Tyr^161^, _QcrB_Thr^313^, and _QcrB_Asp^314^ (Figure 3D) to facilitate electron transfer. In addition, MK could also be modeled to fit deeper inside the pocket (Figure 3—figure supplement 3). Thus, we speculate that what is observed here is the oxidized product as it leaves the Q_P_ site. It is worth noting that all the reported inhibitors including Q203 (Pethe et al., 2013) and TB47 (Lu et al., 2019) are suggested to interact with this Q_P_ site. In addition, the Q_N_ site is formed largely by _QcrB_TMH1, _QcrB_TMH4, _QcrB_TMH5 and one loop region of QcrB (Figure 3E). The head group of MK is bound in this pocket interacting with _QcrB_Phe^39^, _QcrB_Glu^49^, _QcrB_Leu^225^, _QcrB_Leu^232^, _QcrB_Trp^236^, and _QcrB_Phe^262^, and its long hydrophobic tail extends along _QcrB_TMH1 towards the periplasmic side. MK/MKH_2_ are part of the Q-cycle hypothesis and essential for electron transfer in the cytochrome *bcc* complex (Gong et al., 2018). Given the crossspecies activity of this complex (Lee et al., 2020b) and high homology of the QcrB subunits across mycobacterial pathogens (Figure 1), these data open the way for the discovery of broad-spectrum mycobacterial agents based on rational, structure-based inhibitor design principles.

### Q203 interactions in *M. tuberculosis* cytochrome *bcc* binding pocket

**Figure 4.**
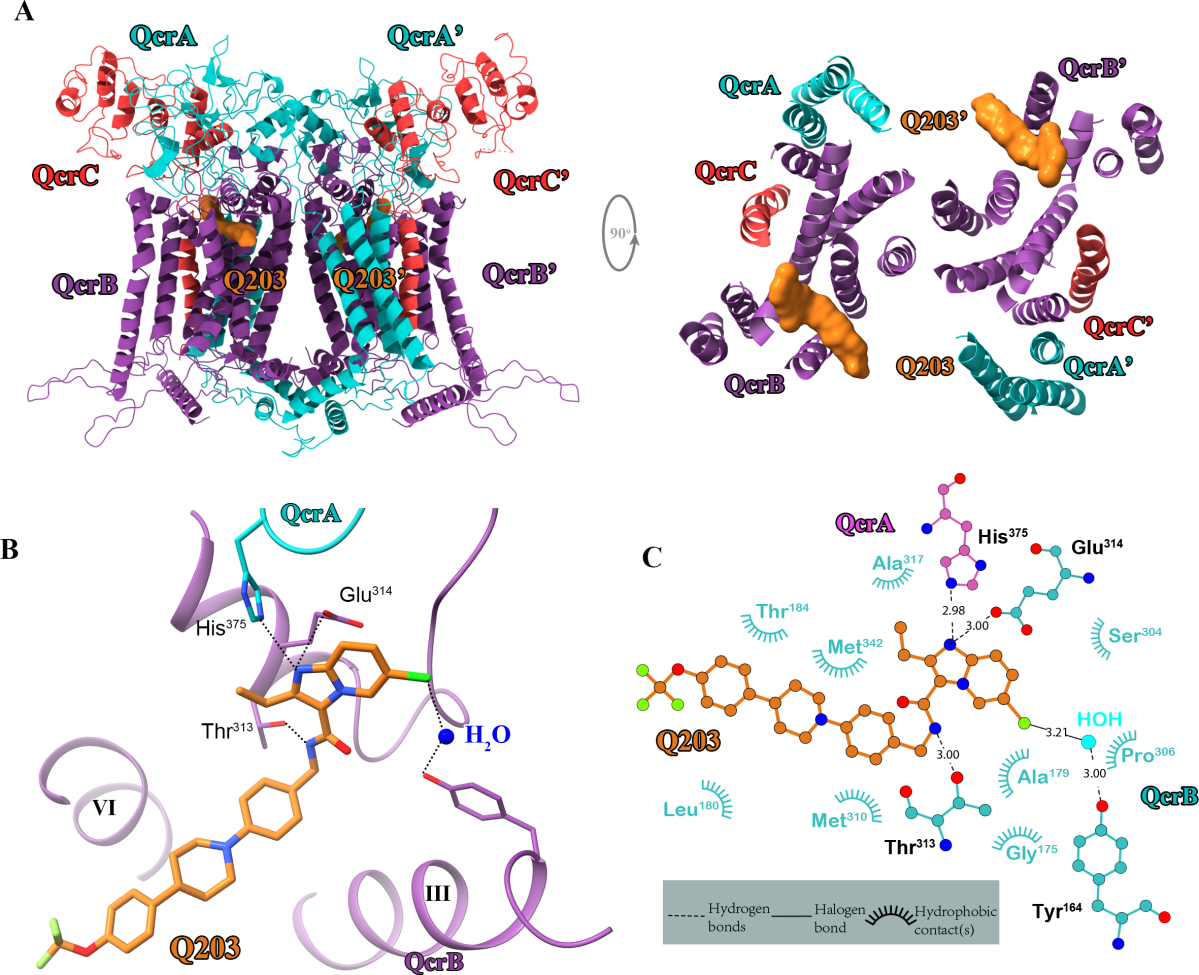
Cryo-electron microscopy (cryo-EM) structure of the hybrid supercomplex in the presence of Q203. (**A**) Side (left) and top (right) views of the cryo-EM structure of the *M. tuberculosis* cytochrome *bcc* complex presented as a cartoon representation. Q203 (orange) is bound to the Qp site. (**B**) Visualization of densities for Q203. Hydrogen bonds are shown as dotted lines. (**C**) Plot of distances of various parts of Q203 to residues in the Qp site as determined using LIGPLOT (https://www.ebi.ac.uk/thornton-srv/software/LIGPLOT/).

Q203 has recently been subjected to a phase II clinical study for *M. tuberculosis* treatment (de Jager et al., 2020). This compound has also been shown to be strongly bactericidal against *M. ulcerans* (Scherr et al., 2018). It is suggested to be an inhibitor that competes with endogenous substrate binding (Q_P_ site) of the cytochrome *bcc* complex (Pethe et al., 2013), but this hypothesis is yet to be verified by direct experimental evidence. To obtain atomic information on the mode of binding of Q203 to cytochrome *bcc*, we have determined the structure of the hybrid supercomplex as described above in the presence of Q203 by cryo-EM to an overall resolution of 2.67 Å (Figure 4—figure supplements 1 and 2, Table 2). We observe that close to the Qp-binding pocket within the membrane of each QcrB of cytochrome *bcc* there is density for Q203 (Figure 4A and B). All of the Q203 molecules fill each QcrB subunit binding deeply into the Q_P_ pocket and with identical binding modes. The key interactions that anchor Q203 are (i) a hydrogen bond between the hydroxyl oxygen of the side chain of _QcrB_Thr^313^ and the amine in the carboxamide linker of Q203 (3.0 Å), (ii) a halogen bond between the chlorine atom of the heterocyclic group and an ordered water molecule that simultaneously forms a hydrogen bond with the hydroxyl oxygen of the side chain of _QcrB_Tyr^164^ (Figure 4—figure supplement 3), (iii) a hydrogen bond between the side chain of _QcrB_Glu^314^ and the nitrogen atom in the imidazopyridine ring (3.0 Å), and (iv) a hydrogen bond between the side chain of _QcrA_His^375^ and the nitrogen atom in the imidazopyridine ring (2.98 Å) (Figure 4C). In addition, the carbon atoms of Q203 make hydrophobic interactions with _QcrB_Gly^175^, _QcrB_Ala^179^, _QcrB_Leu^180^, _QcrB_Thr^184^, _QcrB_Ser^304^, _QcrB_Pro^306^, _QcrB_Met^310^, _QcrB_Ala^317^, and _QcrB_Met^342^. These extensive interactions are in agreement with the fact that the activity of the supercomplex is inhibited by Q203 in vitro according to the DMNQH_2_/oxygen oxidoreductase activity assay. After addition of Q203, the turnover number of the hybrid supercomplex reduces to 5.8 ± 2.4 e^-^s^–1^ from 23.3 ± 2.4 e^-^s^–1^ (mean ± SD, n = 4; Figure 4—figure supplement 4). In addition, functional studies have shown that substitution of _QcrB_Thr^313^ to alanine confers Q203 resistance (Pethe et al., 2013). The binding of Q203 involves residues from both QcrA and QcrB. Due to the need to form stabilizing interactions between subunits, resistance may be more difficult to achieve here than if the binding site is within only one subunit. Consistently, the mapping of reported mutations in Q203-resistant *M. tuberculosis* reveals that they are positioned directly where Q203 binds in this structure (Lupien et al., 2020; Figure 4—figure supplement 5).

**Figure 4—figure supplement 1.**
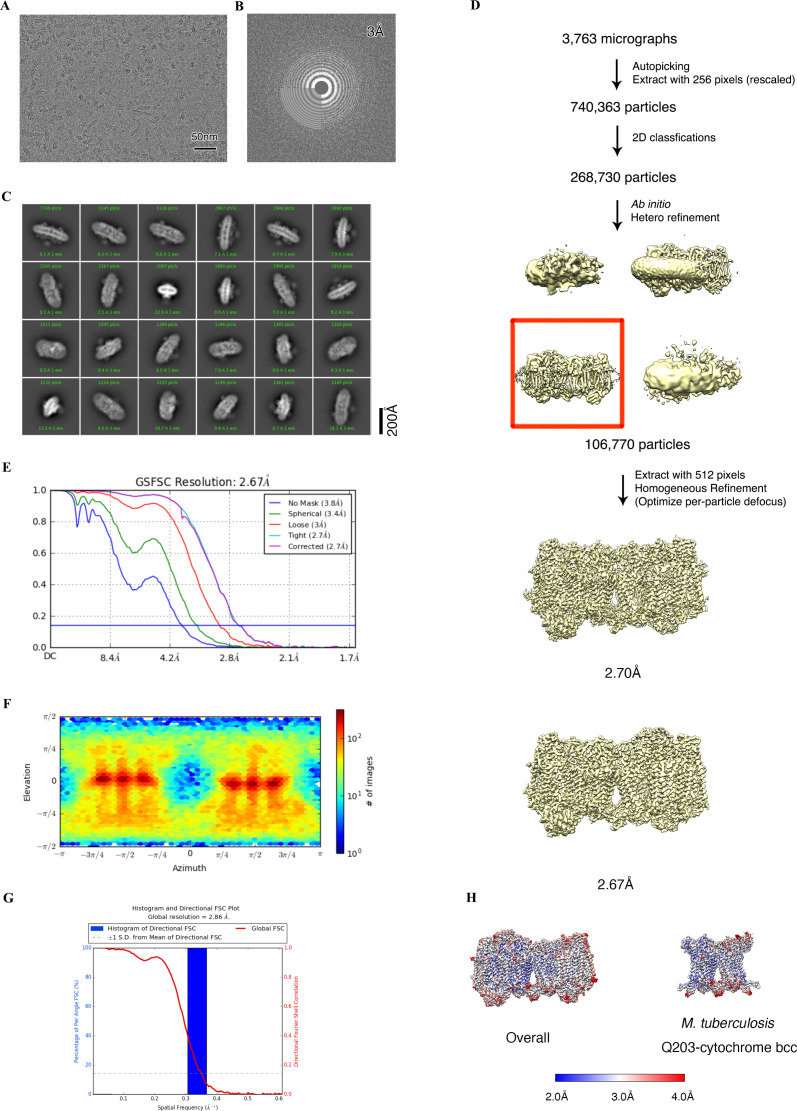
Cryo-electron microscopy (cryo-EM) data processing of the hybrid supercomplex consisting of *M. tuberculosis* CIII and *M. smegmatis* CIV in the presence of Q203. (**A**) Representative electron micrograph of the cryo-EM sample. (**B**) CTF fit of motion-corrected micrographs (**C**) Representative 2D classification averages calculated from selected particles. (**D**) Workflow of data processing for the Q203-bound hybrid supercomplex. (**E**) Fourier shell correlation (FSC) curves of 3D reconstructions. (**F**) Viewing direction of all particles used in the final 3D reconstruction. (**G**) 3D FSC histogram of final map. (**H**) The overall and *Mtb* cytochrome *bcc* maps, colored according to the local resolution.

**Figure 4—figure supplement 2.**
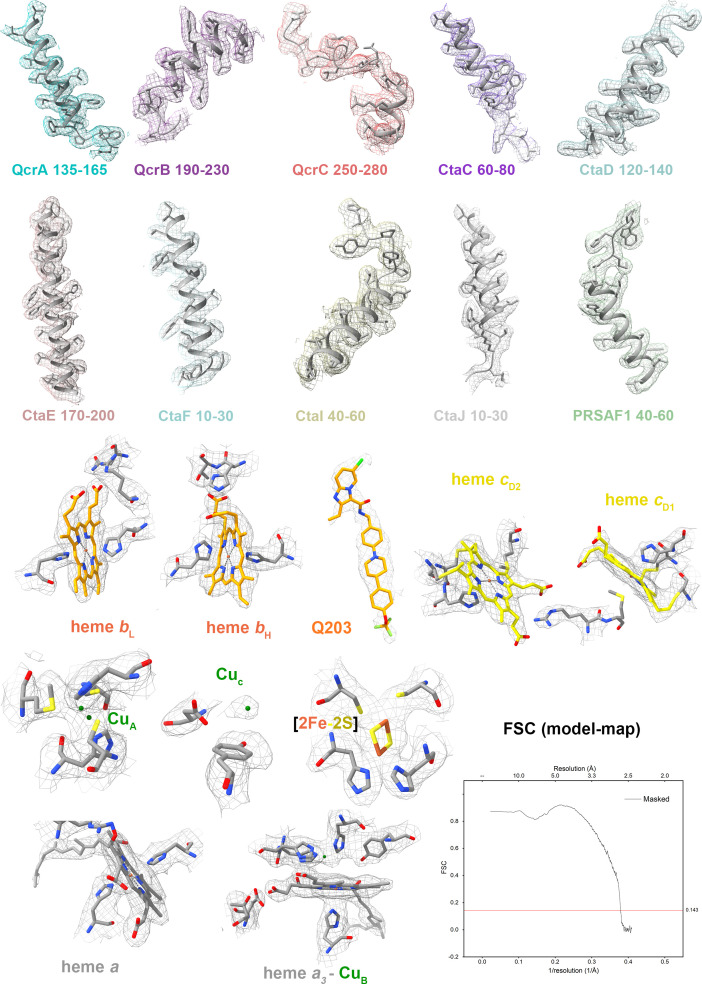
Cryo-electron microscopy (cryo-EM) map quality assessment of *M. tuberculosis* cytochrome *bcc* complex. Representative cryo-EM densities and structures of individual subunits, prosthetic groups, and inhibitors. Map-model Fourier shell correlation (FSC).

**Figure 4—figure supplement 3.**
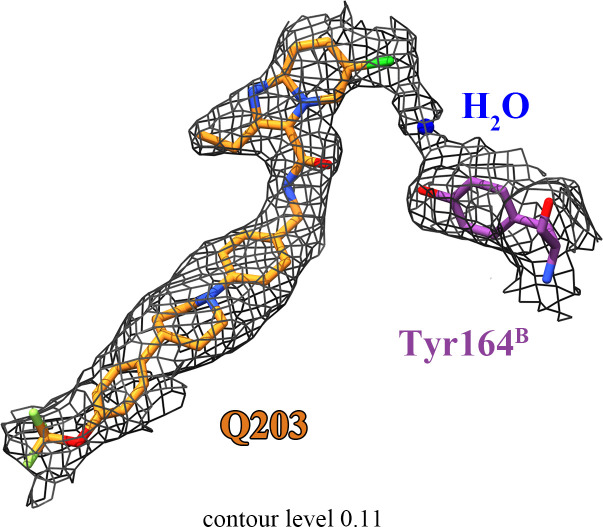
The densities for Q203, H_2_O, and _QcrB_Tyr^164^. Continuous density is observed between the head of Q203, a likely bridging water molecule, and the side chain of _QcrB_Tyr^164^.

**Figure 4—figure supplement 4.**
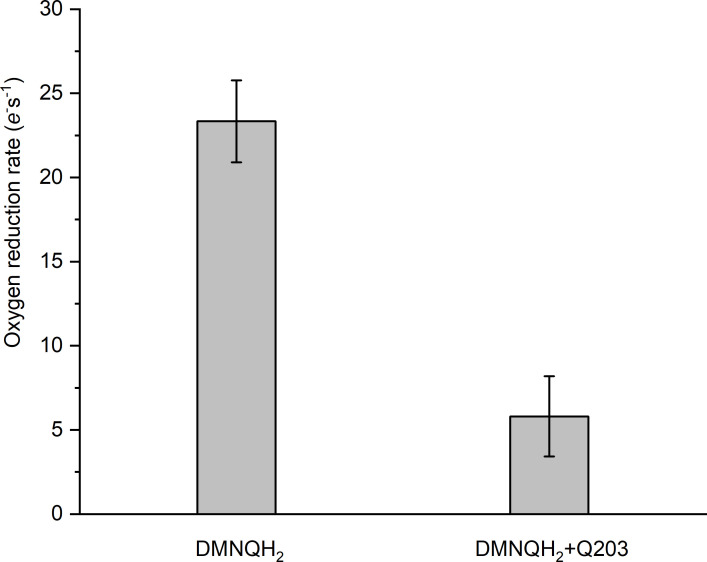
Rate of O_2_ reduction by the hybrid supercomplex before and after addition of Q203. Data shown are mean ± SD; n = 4 measurements. Figure 4—figure supplement 4—source data 1.Oxygen consumption of the hybrid supercomplex after addition of Q203 measures using Clark-type oxygen electrode.

**Figure 4—figure supplement 5.**
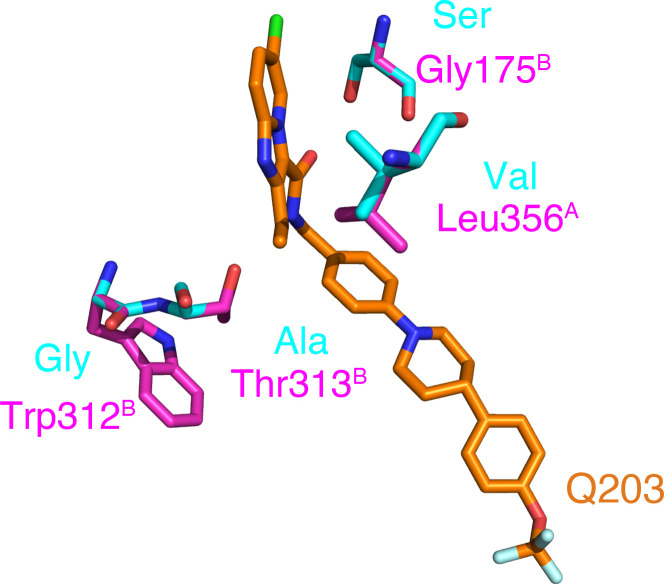
Reported mutations in Q203-resistant *M. tuberculosis*. The native and mutant residues are colored magenta and cyan, respectively.

**Table 2.** Cryo-electron microscopy data collection, refinement, and validation statistics for the Q203-bound hybrid supercomplex.

**State**	**Q203**
*Data collection*	
Microscope	Titan Krios
Voltage (kV)	300
Magnification	29,000×
Detector	Gatan K3
Data collection software	SerialEM
Electron exposure (e^–^/Å^2^)	60
Defocus range (μm)	–1.2 to –1.8
Pixel size (Å)	0.82
*Data processing*	
Number of micrographs	3763
Final particle images	106,770
Symmetry imposed	C1
*Map resolution (Å)*Fourier shell correlation (FSC) 0.143 threshold	
	2.67
*Refinement*	
Initial model used (PDB code)	6ADQ
Map sharpening B factor (Å^2^)d FSC model (0.143) masked	–70.02.6
Map correlation coefficient	0.88
Mean CC for ligands	0.76
*Model composition*	
Non-hydrogen atoms	42,695
Protein residues	5132
Ligands	
	9Y0: 2
	CDL: 17
	9YF: 4
	HEA: 4
	HEC: 4
	MQ9: 8
	HEM: 4
	PLM: 4
	CU: 8
	FES: 2
	HUU (Q203): 2

*Root mean squared deviations*	
Bond lengths (Å)	0.003
Bond angles (°)	0.659
*Validation*	
MolProbity score	1.84
Clashscore	7.97
Poor rotamers (%)	6.23
*Ramachandran plot*	
Favored (%)	93.07
Allowed (%)	6.61
Outliers (%)	0.31
Cβ outliers (%)	0.00

### TB47-binding mode of *M. tuberculosis* cytochrome *bcc*

**Figure 5.**
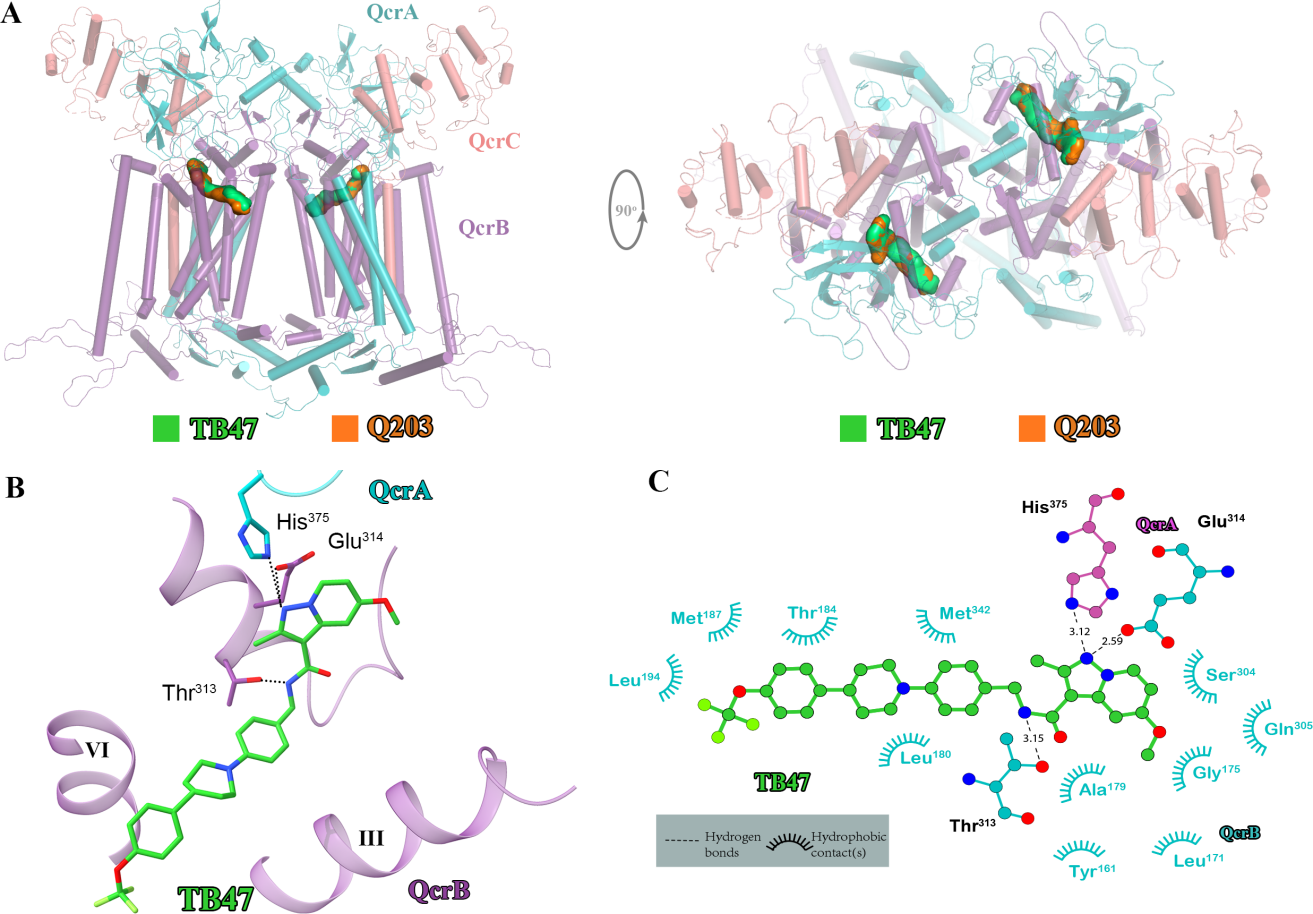
Cryo-electron microscopy (cryo-EM) structure of the hybrid supercomplex in the presence of TB47. (**A**) Side (left) and top (right) views of the cryo-EM structure of the *M. tuberculosis* cytochrome *bcc* complex presented as a cartoon representation. TB47 (green) and Q203 (orange) are bound to the Qp site. (**B**) Visualization of the density for TB47. Hydrogen bonds are shown as dotted lines. (**C**) Plot of distances of various parts of TB47 to residues in the Qp site were determined using LIGPLOT (https://www.ebi.ac.uk/thornton-srv/software/LIGPLOT/).

TB47, also currently being evaluated in preclinical studies, has been suggested to target the QcrB of cytochromes *bcc* from *M. tuberculosis* (Lu et al., 2019) and *M. ulcerans* (Liu et al., 2019). The 2.93 Å cryo-EM map shows density for TB47 and confirms that it binds in the same location as Q203 (Figure 5A and B, Figure 5—figure supplements 1 and 2, Table 3). Three hydrogen bond interactions are observed involving the side chains of _QcrB_Thr^313^, _QcrB_Glu^314^, and _QcrA_His^375^. Similar interactions are also observed when Q203 binds (Figure 5C). _QcrB_Tyr^161^, _QcrB_Leu^171^, _QcrB_Gly^175^, _QcrB_Ala^179^, _QcrB_Leu^180^, _QcrB_Thr^184^, _QcrB_Met^187^, _QcrB_Leu^194^, _QcrB_Ser^304^, _QcrB_Gly^305^, and _QcrB_Met^342^ also contribute to TB47 binding, largely through hydrophobic interactions (Figure 5C). Unlike when Q203 binds (Figure 4—figure supplement 3), there is no interaction between _QcrB_Tyr^164^ and TB47 (Figure 5—figure supplement 3). However, after addition of TB47, the values of turnover number for the hybrid supercomplex reduced to 5.1 ± 2.9 e^-^s^–1^ (mean ± SD, n = 4; Figure 5—figure supplement 4), a similar value that was observed when Q203 binds. Thus, the absence of this does not greatly diminish inhibition. A mutation in TB47-resistant *M. smegmatis* (*M. tuberculosis*: H195Y) is close to the Qp-binding site (Lu et al., 2019; Figure 5—figure supplement 5). As a result of the change in shape, it would appear to contribute to steric interference with the binding of TB47, thus accounting for the observed resistance.

**Figure 5—figure supplement 1.**
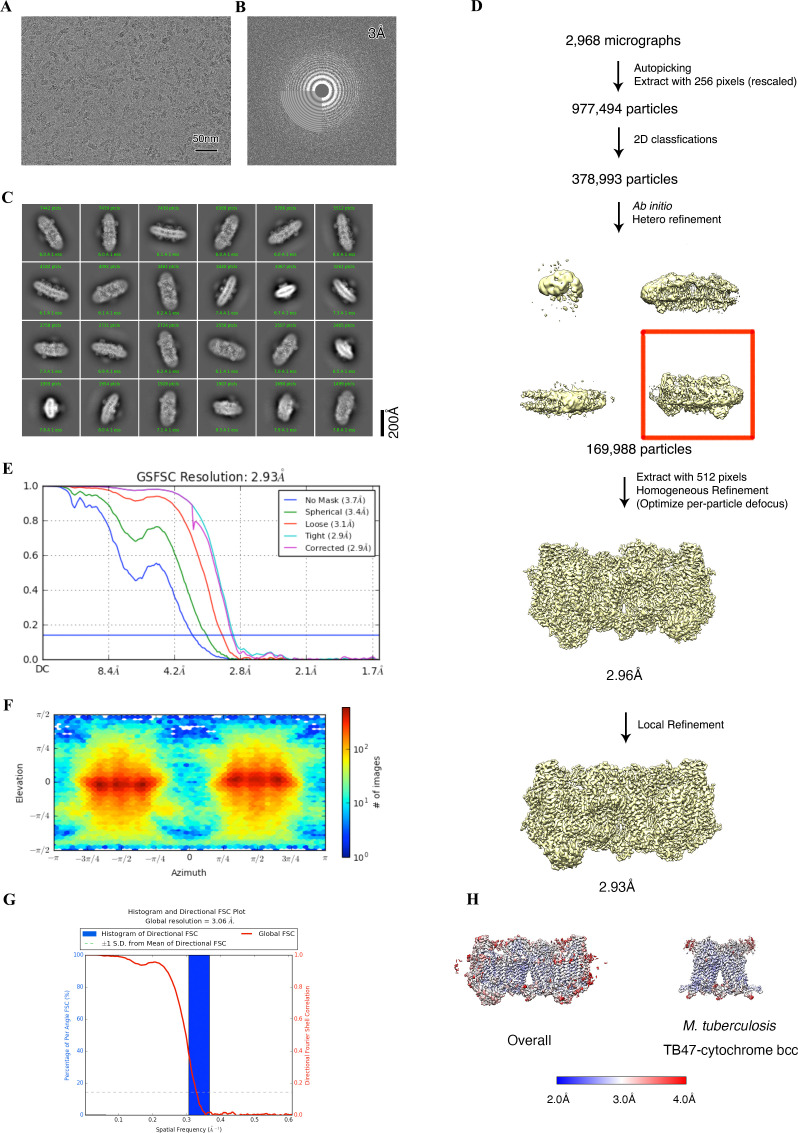
Cryo-electron microscopy (cryo-EM) data processing of the hybrid supercomplex consisting of *M. tuberculosis* CIII and *M. smegmatis* CIV in the presence of TB47. (**A**) Representative electron micrograph of the cryo-EM sample. (**B**) CTF fit of motion-corrected micrographs (**C**) Representative 2D classification averages calculated from selected particles. (**D**) Workflow of the data processing for the TB47-bound hybrid supercomplex. (**E**) Fourier shell correlation (FSC) curves of 3D reconstructions. (**F**) Viewing direction of all particles used in the final 3D reconstruction. (**G**) 3D FSC histogram of final map. (**H**) The overall and *M. tuberculosis* cytochrome *bcc* maps, colored according to the local resolution.

**Figure 5—figure supplement 2.**
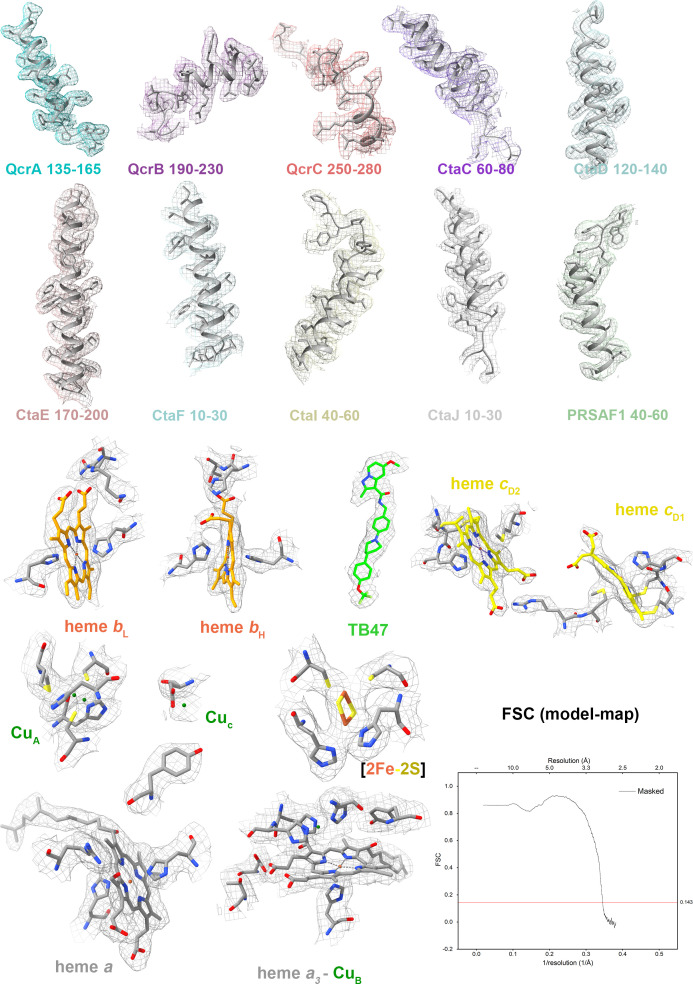
Cryo-electron microscopy (cryo-EM) map quality assessment for the hybrid *M. tuberculosis* cytochrome *bcc* complex. Representative cryo-EM densities and structures of individual subunits, prosthetic groups, and inhibitors. Map-model Fourier shell correlation (FSC).

**Figure 5—figure supplement 3.**
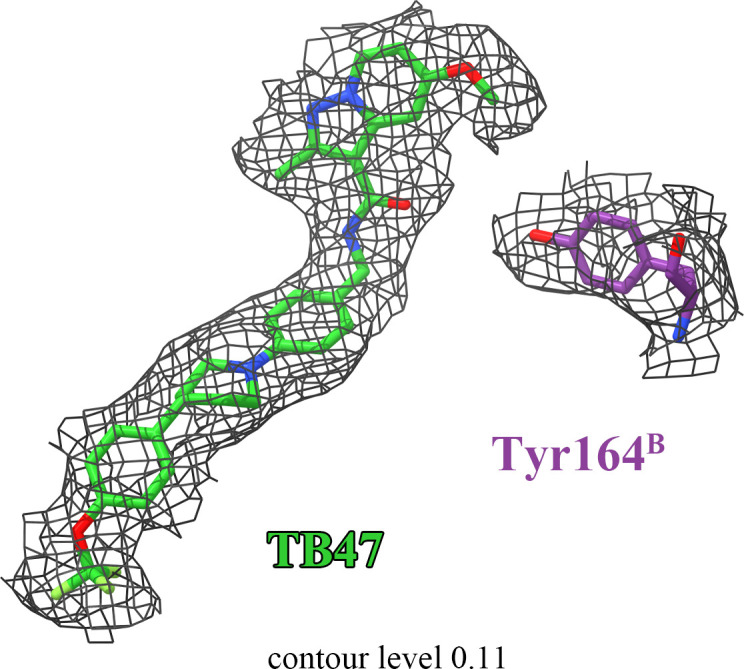
The densities for TB47 and _QcrB_Tyr^164^. There is no density linking TB47 and _QcrB_Tyr^164^.

**Figure 5—figure supplement 4.**
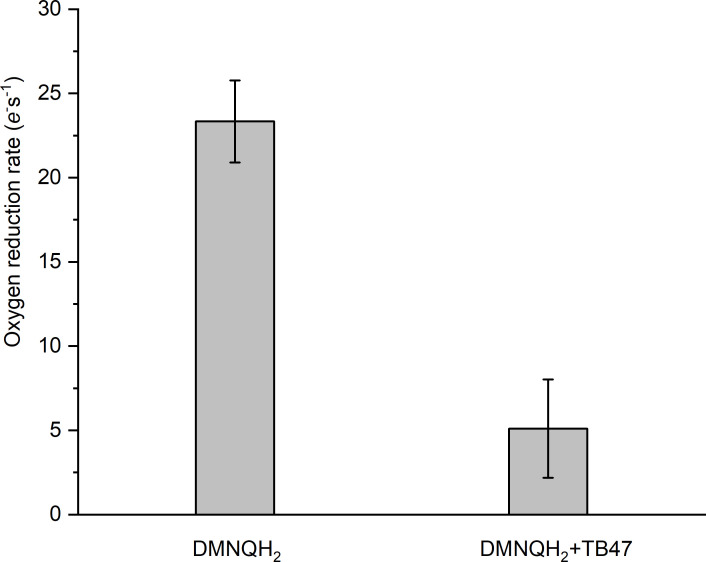
Rate of O_2_ reduction by the hybrid supercomplex before and after addition of TB47. Data shown are mean ± SD; n = 4 measurements. Figure 5—figure supplement 4—source data 1.Oxygen consumption of the hybrid supercomplex after addition of TB47 measures using Clark-type oxygen electrode.

**Figure 5—figure supplement 5.**
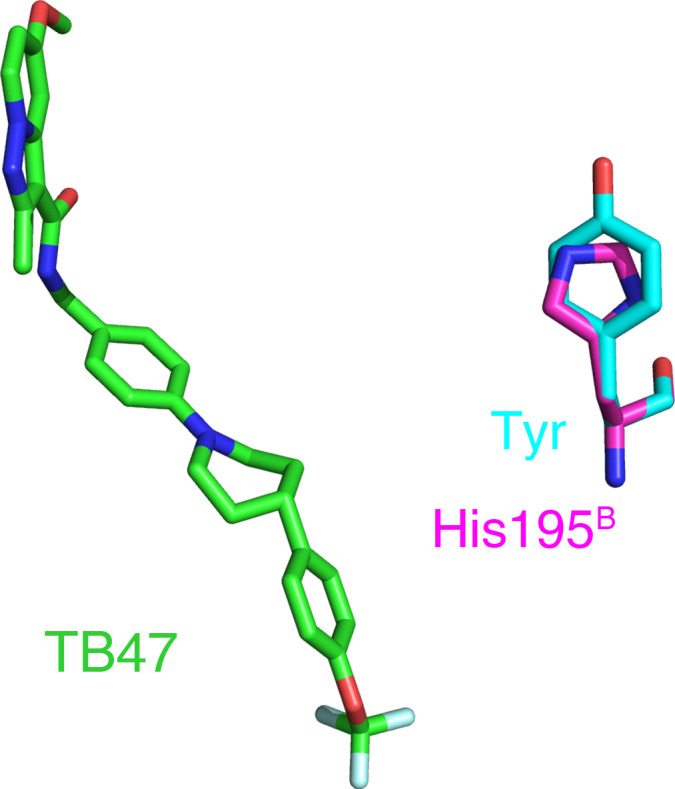
Reported mutations in TB47-resistant *M. tuberculosis*. The native and mutant residues are colored magenta and cyan, respectively.

**Table 3.** Cryo-electron microscopy data collection, refinement, and validation statistics for the TB47-bound hybrid supercomplex.

**State**	**TB47**
*Data collection*	
Microscope	Titan Krios
Voltage (kV)	300
Magnification	29,000×
Detector	Gatan K3
Data collection software	SerialEM
Electron exposure (e^–^/Å^2^)	60
Defocus range (μm)	–1.2 to –1.8
Pixel size (Å)	0.82
*Data processing*	
Number of micrographs	2698
Final particle images	169,988
Symmetry imposed	C1
*Map resolution (Å)*Fourier shell correlation (FSC) 0.143 threshold	
	2.93
*Refinement*	
Initial model used (PDB code)	6ADQ
Map sharpening B factor (Å^2^)d FSC model (0.143) masked	–97.52.9
Map correlation coefficient	0.90
Mean CC for ligands	0.79
*Model composition*	
Non-hydrogen atoms	42,679
Protein residues	5119
Ligands	
	9Y0: 2
	CDL: 17
	9YF: 4
	HEA: 4
	HEC: 4
	MQ9: 8
	HEM: 4
	PLM: 4
	CU: 8
	FES: 2
	HV0 (TB47): 2

*Root mean squared deviations*	
Bond lengths (Å)	0.005
Bond angles (°)	0.739
*Validation*	
MolProbity score	1.87
Clashscore	8.75
Poor rotamers (%)	6.23
*Ramachandran plot*	
Favored (%)	92.39
Allowed (%)	7.25
Outliers (%)	0.36
Cβ outliers (%)	0.00

### Specificity of Q203 and TB47 for mycobacterial cytochrome *bcc* complex

**Figure 6.**
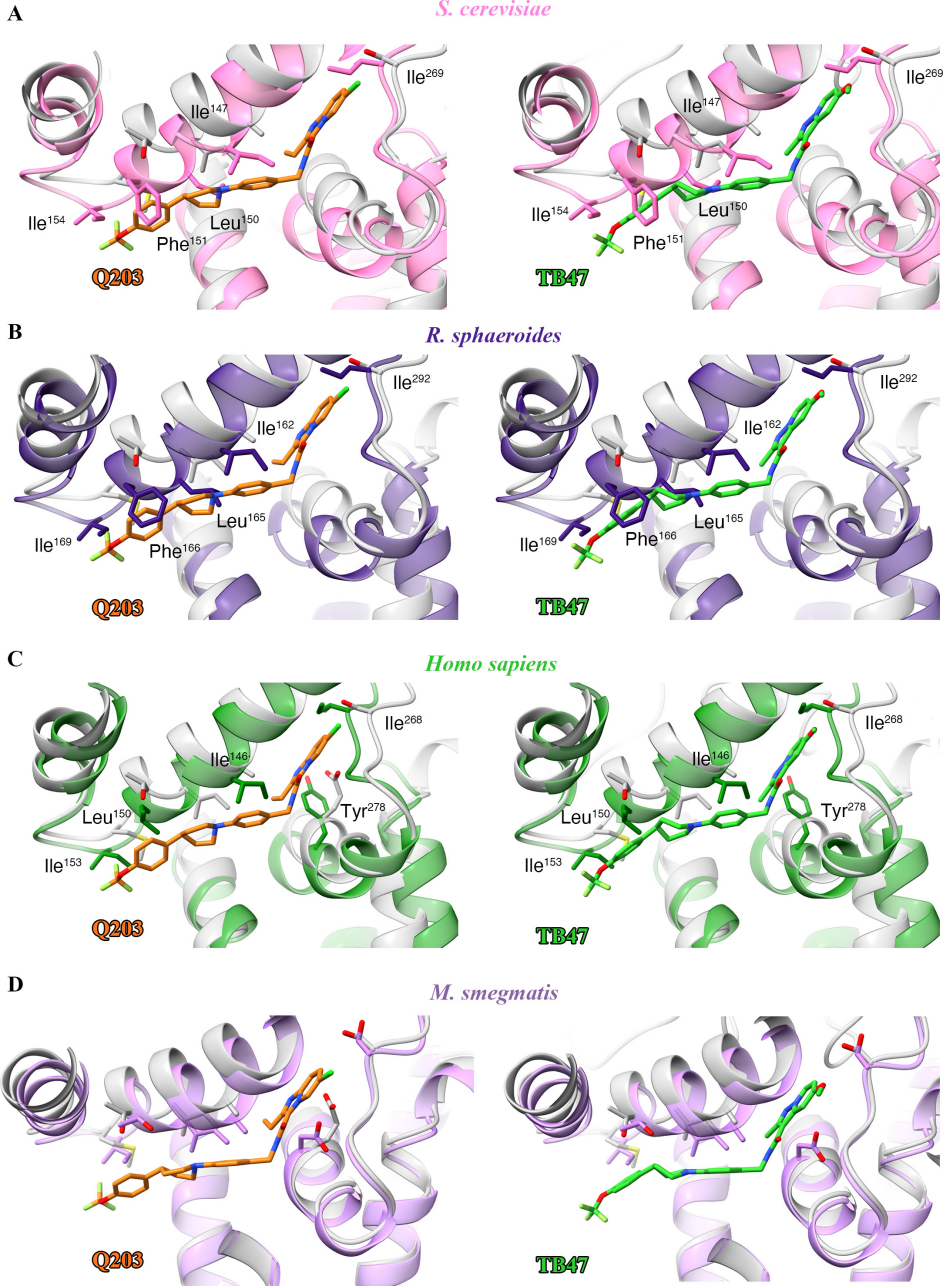
Structural alignment between the *M. tuberculosis* Qp-binding pocket where Q203 or TB47 binds with homologous subunits from four other species. These subunits are from (**A**) *S. cerevisiae* (pink, PDB: 1KYO), (**B**) *R. sphaeroides* (blue, PDB: 2QJP), (**C**) *Homo sapiens* (green; PDB: 5XTE), and (**D**) *M. smegmatis* (violet, PDB: 6ADQ). Residues causing steric clashes in the homologous subunits are labeled.

The basis for the high specificity of Q203 and TB47 toward the Qp site of mycobacterial cytochromes *bcc* becomes apparent in the structural comparison between the QcrB subunit of *M. tuberculosis* and counterparts from other species (Figure 6). The highly conserved residues that are involved in the binding of these two molecules in this region (Figure 6—figure supplement 1) suggest a consistent overall fold and binding site exists in mycobacteria. This is also in agreement with the fact that Q203 and TB47 show antimycobacterial activity across many species (de Jager et al., 2020; Liu et al., 2019; Lu et al., 2019; Pethe et al., 2013; Scherr et al., 2018). It is worth noting that the structures of cytochrome *bcc* from *M. tuberculosis* and *M. smegmatis* have high similarity (Figure 2—figure supplement 5), and no steric hindrance is observed between the Q203 and *M. smegmatis* cytochrome *bcc* (Figure 6). This observation indicates that Q203 should have a similar binding mechanism and a similar effect on the activity of cytochrome *bcc* from *M. smegmatis* and *M. tuberculosis*. This is in good agreement with previous antimycobacterial activity data and inhibition data for the *bcc* complexes from *M. smegmatis* and *M. tuberculosis* (Gong et al., 2018; Lu et al., 2018). In contrast, in other prokaryotic, eukaryotic and human Qp-binding pockets, for example, from *Saccharomyces cerevisiae* (Lange and Hunte, 2002), *Rhodobacter sphaeroides* (Esser et al., 2008), and human (Guo et al., 2017), many of the observed interactions would not be possible (Figure 6). This suggests that Q203 and TB47 should have low-binding affinity toward its counterpart QcrB in non-mycobacterial bacteria and in eukaryotes. Even if there is some flexibility in the Qp-binding pocket that enables some level of binding, key residues that enable the binding of Q203 and TB47 in the mycobacteria are not present in other bacteria and eukaryotes (Figure 6—figure supplement 1). In addition, the binding free energies of Q203 between wild-type and mutant hybrid supercomplex were calculated and used to further elucidate the importance of some key residues that could play a role in determining the inhibitor specificity. According to a previous functional study (Pethe et al., 2013), structural comparison (Figure 6), and sequence alignment (Figure 6—figure supplement 1), _QcrB_Thr^313^ and _QcrB_Glu^314^ of hybrid supercomplex were selected and mutated to their counterparts in the human supercomplex, namely residues Ala and Tyr, respectively. It is believed that Ala would decrease the affinity of Q203 (Figure 4; Pethe et al., 2013) and the Tyr could lead to steric hindrance of Q203 binding (Figure 6). 75 ns simulations were used for the calculation of binding free energy (Figure 6—figure supplement 2). The different values for the root mean squared deviation (RMSD) suggest that three mutants might have the different effects on the structural conformation of QcrB, and the dual mutant (Thr313Ala and Glu314Tyr) induced significantly structural changes compared to the wild-type complex. The 75 ns simulation was further equally divided into three phases, and the average values were used as the final relative binding free energy (Table 4), which was calculated as the binding free energy of Q203 in the mutant minus that of the wild-type systems. It is suggested that the wild-type system has stronger affinity than the mutant systems (Thr313Ala or Glu314Tyr). The relative free energy of binding calculation for Q203 in the dual mutant (Thr313Ala and Glu314Tyr) indicates that its binding affinity is significantly weaker compared to the wild-type or single-mutant systems. Therefore, the suggestions drawn from the calculation of binding free energy of Q203 provide further evidence for specificity of Q203 inhibition. In summary, these observations correlate with the observed low general antibacterial activity and low cytotoxicity of Q203 and TB47 (Liu et al., 2019; Lu et al., 2019; Pethe et al., 2013; Scherr et al., 2018).

**Figure 6—figure supplement 1.**
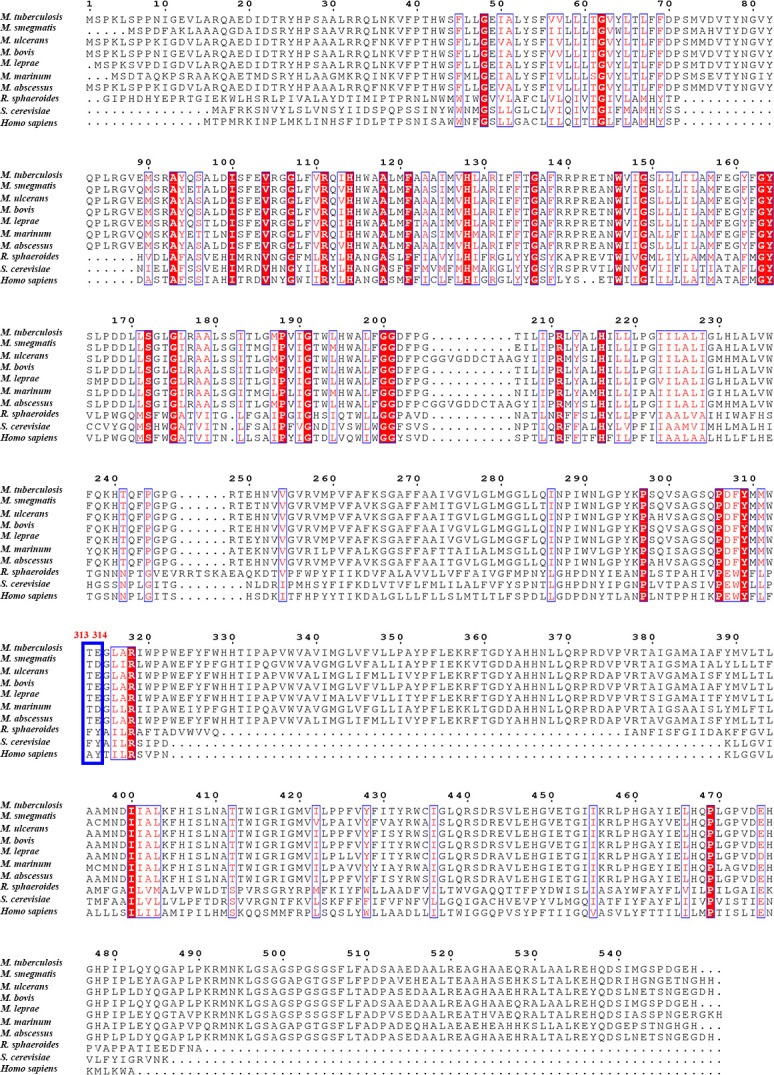
Sequence alignment of *M. tuberculosis* QcrB with their counterparts in other species including *Homo sapiens*. Red residues are conserved, and blue indicates those less well conserved.

**Figure 6—figure supplement 2.**
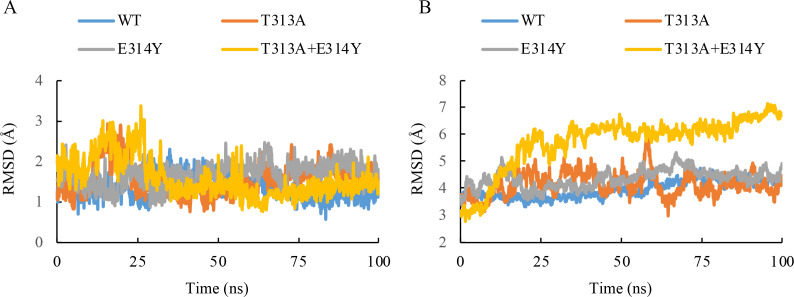
Molecular dynamics simulation plot for the root mean squared deviation (RMSD) of the heavy atoms in the inhibitor (**A**) and main chain atoms of QcrB (**B**). 100 ns NPT simulations for the wild-type and three mutant systems of the Q203-bound QcrB complex (T313A, E314Y, T313A + E314Y) are recorded. The frames were extracted from the 100 ns simulation every 100 ps, generating 1000 frames. The RMSD values of each frame were calculated based on the structural conformation of the reference structures, namely the initial wild-type and mutant Q203-bound QcrB complexes, respectively. Figure 6—figure supplement 2—source data 1.Root mean squared deviation of the inhibitor and protein of Q203-bound QcrB complex.

**Table 4.** Relative binding free energy (kcal/mol) for Q203 in three mutants of QcrB compared to the wild-type (WT).

Mutant	25–50 ns	50–75 ns	75–100 ns	Average	Standard deviation
T313A	6.32	11.01	8.11	8.48	2.37
T314Y	4.54	6.00	8.07	6.20	1.77
T313A + E314Y	10.28	16.30	13.64	13.41	3.02

### Implications of the Q203 and TB47 inhibitory mechanism

To gain further insights into the mechanism of action of Q203, we compared the structures of *M. tuberculosis* cytochrome *bcc* in the presence and absence of Q203 (Figure 7—figure supplement 1A). The structure of apo cytochrome *bcc* is almost identical with the Q203-bound structure (rmsd of 0.50 Å for all Ca atoms), which suggests that Q203 binding does not significantly affect the overall architecture of cytochrome *bcc*. A comparison of the Q203-bound and Q203-free cytochrome *bcc* structures shows residues involved in the binding pocket move outward, thus adapting to the shape of Q203 (Figure 7—figure supplement 1B). Specifically, the side chains of _QcrB_Ser^304^, _QcrB_Glu^313^, _QcrB_Glu^314^, and _QcrB_Met^342^ undergo significant conformational changes to form hydrogen bonds with Q203. The binding of TB47 to *M. tuberculosis* cytochrome *bcc* also induces very similar conformational changes in the Qp-binding pocket to those seen for Q203 (rmsd of 0.454 Å for all Ca atoms) (Figure 7—figure supplement 1B). Differences in binding are due to the different ethyl group and methyl moieties in the head groups of Q203 and TB47. It is also important to note that one endogenous substrate molecule is also bound to the Qp site in the apo structure of cytochrome *bcc*, which potentially affects the evaluation of the conformational changes upon the binding of Q203 or TB47.

**Figure 7.**
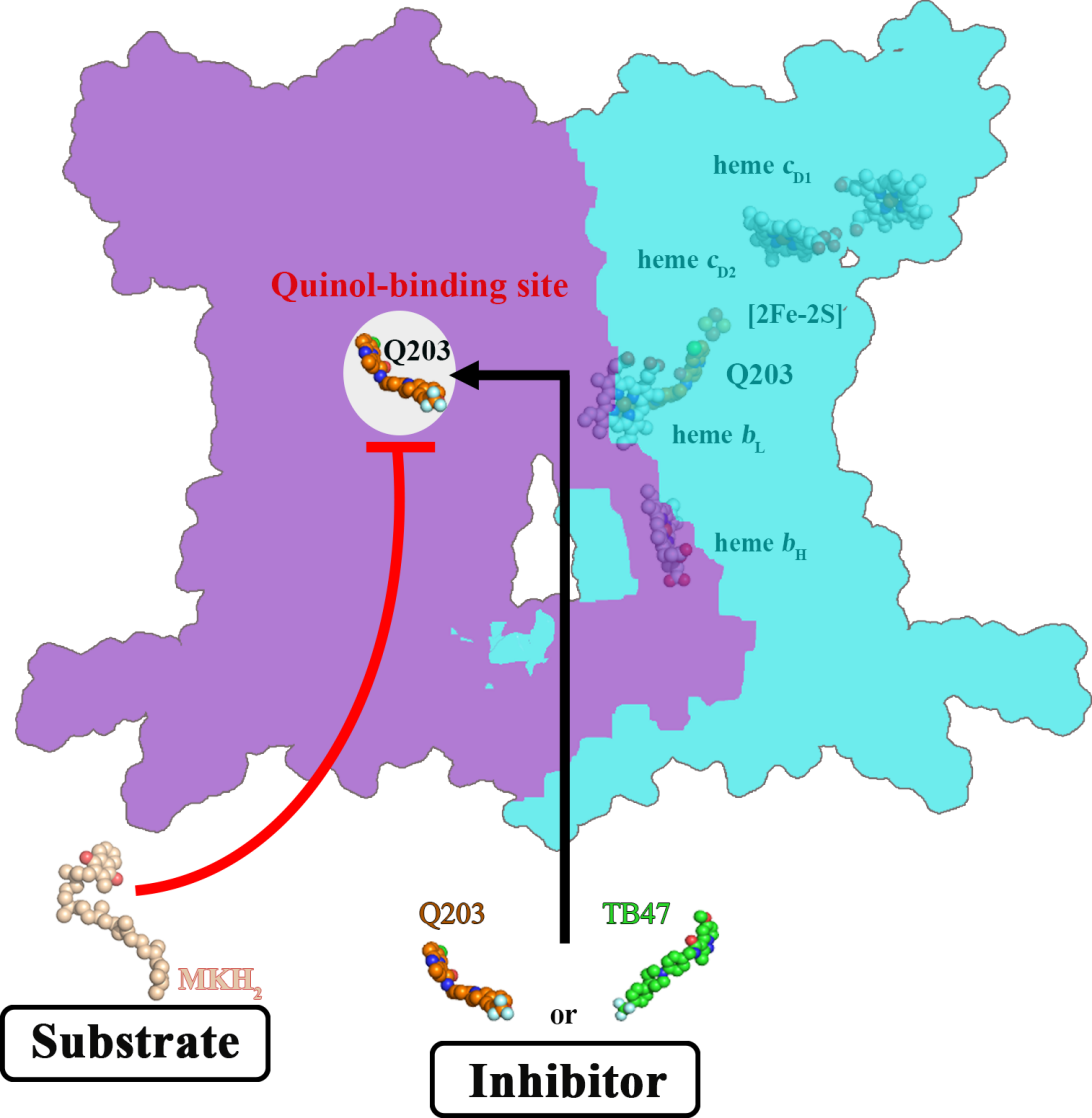
Schematic of *M. tuberculosis* cytochrome *bcc* inhibition by Q203 and TB47. The two monomers of *M. tuberculosis* cytochrome *bcc* are colored magenta and cyan, respectively. The binding of Q203 (orange spheres) or TB47 (green spheres) prevents substrate access (gray spheres).

When analyzing the superimposed structures (Figure 7—figure supplement 1), it is apparent that Q203 and TB47 act competitively with the quinol binding as they almost completely occupy the Qp pocket. We therefore conclude that Q203 and TB47 are bona fide analogs of the substrate, and thus ultimately function by hindering the downstream synthesis of ATP (Figure 7). These two compounds are also highly bactericidal against *M. ulcerans*, almost certainly targeting the Qp-binding site (Liu et al., 2019; Scherr et al., 2018). In summary, the sequences of the QcrB subunits have high homology across pathogenic mycobacteria (Lee et al., 2020b) and the essential residues (_QcrB_Glu^313^ and _QcrB_Glu^314^) that are involved in hydrogen-bonding interactions with the inhibitors (Pethe et al., 2013; Scherr et al., 2018) are conserved across pathogenic mycobacteria (Figure 7—figure supplement 2).

**Figure 7—figure supplement 1.**
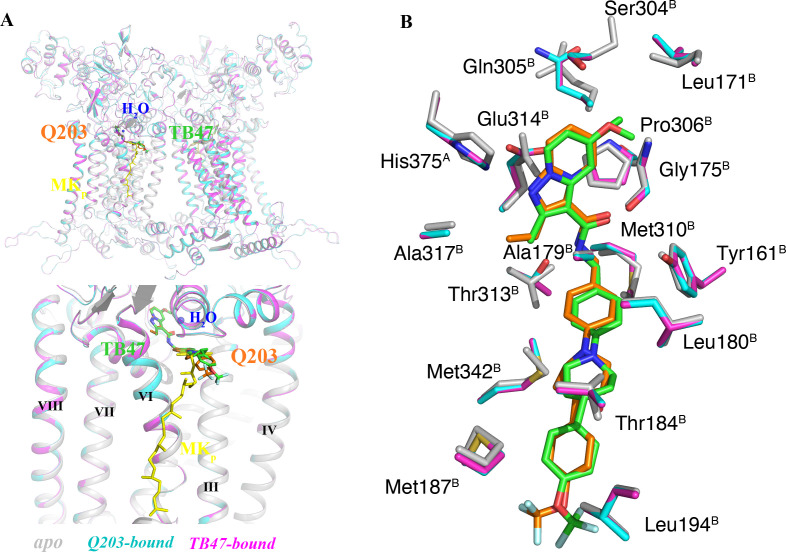
Comparison of apo and Q203/TB47-bound structures of *M. tuberculosis* cytochromes *bcc*. (**A**) Superposition of apo (gray), Q203-bound (cyan), and TB47-bound (magenta) structures of *M. tuberculosis* cytochromes *bcc*. The Q203 (orange), TB47 (green), and MK (yellow) molecules are shown as stick models, respectively. Water molecules are shown as spheres. (**B**) Comparison of residues surrounding Q203 (cyan sticks) and TB47 (magenta stick models) with those in apo form (gray sticks). The residues from subunits A and B are labeled with superscript A and B, respectively.

**Figure 7—figure supplement 2.**
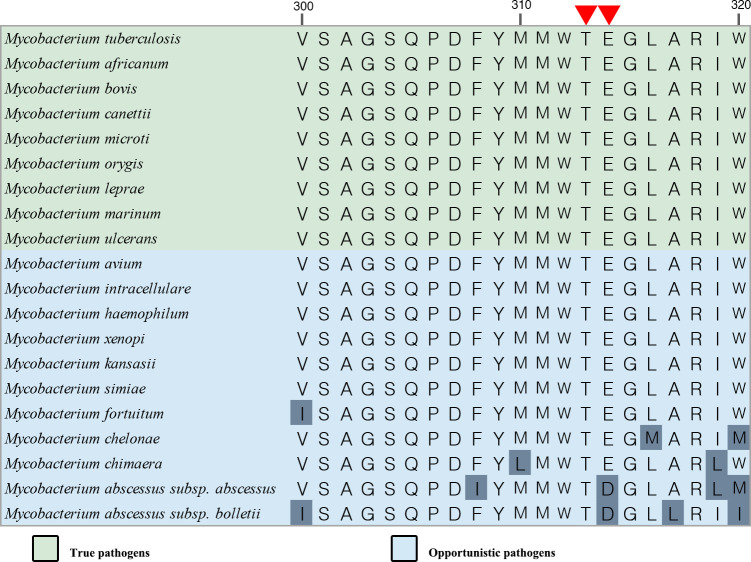
Sequence alignment for the Q_P_ site within pathogenic mycobacteria. Partial sequence alignment at the Qp site. The red triangles indicate the conservative residues involved with the hydrogen-bond formation between Q203 and TB47.

### Conclusions

We have determined the apo- and Q203 and Tb47-bound structures of a hybrid pathogenic *M. tuberculosis/M. smegmatis* cytochrome *bcc* complex. The study shows the structural features of *M. tuberculosis* cytochrome *bcc* and how it is specifically inhibited by Q203 and TB47. The extensive interactions between Q203 or TB47 and the Qp-binding pocket account for the highly specific binding of these two inhibitors to pathogenic *M. tuberculosis* cytochrome *bcc* compared to eukaryotic counterparts. Two conservative residues involved with the formation of hydrogen bonds are observed across the pathogenic mycobacteria. These structures provide a long-sought basis for rational, structure-based inhibitor design to accelerate the development of Q203 and TB47 analogs as drug leads for mycobacterial infections.

## Materials and methods

**Key resources table:** 

Reagent type (species) or resource	Designation	Source or reference	Identifiers	Additional information
Gene (*Mycobacterium tuberculosis*)	qcrC	Mycobrowser	Rv2194	https://mycobrowser.epfl.ch/genes/Rv2194
Gene (*M. tuberculosis*)	qcrA	Mycobrowser	Rv2195	https://mycobrowser.epfl.ch/genes/Rv2195
Gene (*M. tuberculosis*)	qcrB	Mycobrowser	Rv2196	https://mycobrowser.epfl.ch/genes/Rv2196
Strain, strain background (*Mycobacterium smegmatis*)	mc^2^ 51	Li et al., 2014		
Genetic reagent (include species here)	pVV16-QcrCAB-His10	This paper		Construct contains the qcrCAB operon encoding three subunits
Commercial assay or kit	ClonExpress II One Step Cloning Kit	Vazyme	C112-01	
Chemical compound, drug	Q203	MCE	HY-101040	Prepare stock solution in DMSO
Chemical compound, drug	TB47	Lu et al., 2019		Prepare stock solution in DMSO
Chemical compound, drug	LMNG	Anatrace	NG310	
Chemical compound, drug	Digitonin	BIOSYNTH	D-3200	
Software, algorithm	SerialEM	Mastronarde, 2003		Version 3.6
Software, algorithm	MotionCor2	Zheng et al., 2017		Version 1.2.1
Software, algorithm	RELION	Zivanov et al., 2019		Version 3.03
Software, algorithm	cryoSPARC	Punjani et al., 2017		Version 3.2.0
Software, algorithm	Phyre2	Kelley et al., 2015		Version 2.0
Software, algorithm	UCSF Chimera	Pettersen et al., 2004		Version 1.12
Software, algorithm	COOT	Emsley et al., 2010		Version 0.8.9
Software, algorithm	PHENIX	Adams et al., 2010		Version 1.16
Software, algorithm	PyMOL	Schrödinger LLC, 2017		Version 2.0
Software, algorithm	ChemDraw	Li et al., 2004		Version 19.0

### Expression of hybrid supercomplex consisting of *M. tuberculosis* CIII and *M. smegmatis* CIV

The hybrid supercomplex was obtained according to a previous study (Kim et al., 2015) but with some modifications. The *M. tuberculosis* cytochrome *bcc* complex is encoded by three putative genes (Rv2194-2196). Genes were amplified from H37Rv genomic DNA by PCR using Phanta Max DNA polymerase (Vazyme), and two-step PCR was used to inset a 10× His tag at the C-terminus of the qcrB (Rv2196). Genes encoding the entire cytochrome *bcc* complex operon were then cloned into the pVV16 expression vector. The resultant plasmid was transformed into *M. smegmatis* mc^2^ 51 (Li et al., 2014) cells whose qcrCAB operon encoding *M. smegmatis* cytochrome *bcc* had already been knocked out. The cells were cultivated in Luria-Bertani broth (LB) liquid media supplemented with 50 μg/mL hygromycin, 25 μg/mL streptomycin, and 0.1% Tween 80. Cell pellets were harvested by centrifugation when the cells were grown to an optical density (OD_600_ of 1.0–1.2) at 37°C (220 rpm). Harvested cells were frozen at –80°C until use.

### Purification of the hybrid supercomplex

Cell pellets were thawed and resuspended in buffer A containing 20 mM 3-(N-morpholino)propanesulfonic acid (MOPS), pH 7.4, 100 mM NaCl, and then lysed by passing through a French Press at 1200 bar three times. Cell debris and non-lysed cells were removed by centrifugation at 14,000 rpm for 10 min at 4°C. The supernatant was collected and ultra-centrifuged at 36,900 rpm and 4°C for 2 hr. The membrane fraction was solubilized by addition of 1% (w/v) lauryl maltose neopentyl glycol (LMNG) in buffer A and incubated for 2 hr at 4°C with slow stirring. The suspension was ultra-centrifuged, and the supernatant was applied to Ni-NTA agarose beads (GE Healthcare) at 4°C. The beads were further washed in buffer A with 50 mM imidazole and 0.004% (w/v) LMNG. The buffer was exchanged to buffer B (20 mM MOPS, pH 7.4, 100 mM NaCl, and 0.1% [w/v] digitonin) and then washed in resin in batch mode. The protein was eluted from the beads with buffer B containing 500 mM imidazole. Protein was then concentrated and loaded onto a Superdex 6 increase (10/300GL, GE Healthcare) column equilibrated in buffer B. Peak fractions were pooled and concentrated to ~8 mg/mL for electron microscopy studies. The protein sample was analyzed by sodium dodecyl sulfate polyacrylamide gel electrophoresis (SDS-PAGE), and the bands were then identified through mass spectrometry.

### Activity assays

2,3-Dimethyl-1,4-naphthoquinone (DMNQ, CAS 2197-57-1) was synthesized by WuXi AppTec. In order to obtained reduced DMNQH_2_, 20 mM DMNQ was ultrasonically dissolved in 1 mL ethanol with 6 mM HCl and reduced with sodium borohydride (NaBH_4_) in the ice bath. An appropriate amount of 12 N HCl was added into quench unreacted NaBH_4_ under the protection of argon. Oxidase activity was determined by a method described previously (Bonner et al., 1986; Kusumoto et al., 2000; Safarian et al., 2019). The oxygen consumption was monitored using the Clark-type oxygen electrode (Oxytherm^+^, Hansatech) in 1 mL reaction buffer (20 mM MOPS, pH 7.4, 100 mM NaCl, 0.004% LMNG) at 25℃, containing 62.5 nM hybrid supercomplex. The reaction was started by addition of 100 μM DMNQH_2_. The oxygen consumption curve was plotted using GraphPad Prime 8.0 software, from which an estimate of the oxygen-reduction rate (*v*_0_) was obtained (corrected for autoxidation). In the inhibition assay, 500 nM Q203 or TB47, which is approximately the value of the median inhibitory concentration (IC50) according to our previous study (Gong et al., 2018), was chosen and incubated with 62.5 nM hybrid supercomplex for 20 min at 25℃. Inhibition curves and oxygen-reduction rates (*v*_i_) with the different inhibitors were recorded. The inhibition rate (1 *v*_0_/*v*_i_) of Q203 and TB47 at 500 nM is reported. This assay was conducted using four groups of parallel experiments.

### Cryo sample preparation and data collection

300-mesh Quantifoil R0.6/1.0 grids (Quantifoil, Micro Tools GmbH, Germany) were glow-discharged at H_2_/O_2_ atmosphere for 25 s. 3 μL aliquots of protein complex at a concentration of 10 mg/mL were applied to the grid and then blotted for 3 s with force 0 at 8°C and 100% humidity using a Vitrobot IV (Thermo). Images were collected using a Titan Krios 300 keV electron microscope (Thermo), equipped with K3 Summit direct electron detector camera (Gatan). Data were recorded at 29,000× magnification with a calibrated super-resolution pixel size 0.82 Å/pixel. The exposure time was set to 2.4 s with 40 subframes and a total dose of 60 electrons per Å^2^. All images were automatically recorded using SerialEM with a defocus range from 1.2 μm to 1.8 μm (Mastronarde, 2003). For the datasets of apo, Q203-bound and TB47-bound *M. tuberculosis* cytochrome *bcc*, a total of 4141, 3763, and 2968 images were collected, respectively.

### Image processing

All dose-fractioned stacks were motion-corrected and dose-weighted using MotionCorr2 (Zheng et al., 2017) in RELION 3.03 (Zivanov et al., 2019). CTF estimation was conducted using cryoSPARC patch CTF estimation (Punjani et al., 2017). For the dataset of apo hybrid *M. tuberculosis* cytochrome *bcc*, 1,208,054 particles were picked automatically using EMD-9610 map as the template and extracted with a box size of rescaled 256 pixels (binned 2). 327,188 particles were selected after two rounds of 2D classification. 100,000 particles were used to perform *ab initio* reconstruction in four classes, and these four classes were used as 3D volume templates for heterogeneous refinement with all selected particles. 112,804 particles converged into one class with clear signals and then re-extracted with 512 pixels (binned 1). Next, this particle set was used to do homogeneous refinement and local refinement, yielding the final resolution 2.68 Å. For the dataset of Q203-bound and TB47-bound *M. tuberculosis* cytochrome *bcc*, the data processing was performed in a similar pipeline, resulting in the final reconstruction resolution at 2.67 Å and 2.93 Å, respectively (detailed parameters shown in supplementary figures).

### Model building and validation

The *M. smegmatis* respiratory complex CIII_2_CIV_2_ (PDB code: 6ADQ) model (Gong et al., 2018) as rigid body was fitted into EM density maps using UCSF Chimera 1.12 (Pettersen et al., 2004). Next, the resultant atomic model was manually modified according to the subunit sequences of *M. tuberculosis* cytochrome *bcc* and refined in COOT 0.8.9.1 (Emsley et al., 2010), followed by real-space refinement in PHENIX (Adams et al., 2010). The smile strings of Q203 and TB47 were generated and copied from ChemDraw (Li et al., 2004) and defined in PHENIX elBOW. Q203 and TB47 were manually built into the corresponding EM densities. The local resolution map was calculated in cryoSPARC (Punjani et al., 2017). All reported resolutions were based on the gold-standard FSC 0.143 criteria (Rosenthal and Henderson, 2003).

### MK9 docking study

The binding pose of MK9 with QcrB was generated by molecular docking using the Glide (Schrödinger, LLC). The spatially neighboring subunits QcrA/B were extracted from the hybrid supercomplex and used as the model. The protein structure was processed using the Protein Preparation Wizard module. In this process, hydrogens were added to heavy atoms and bond orders were assigned to each bond. The protonation state of each amino acid was predicted at pH 7.0 using the Epik algorithm (Shelley et al., 2007). All resolved waters were removed from the structure. At the same time, the structure of MK9 was prepared using the LigPrep module, and a low-energy conformation of MK9 was generated. The binding pocket of Q203 in QcrB was defined as the docking site using the Receptor Grid Generation module. The docking site was confined to an enclosed cube with side length of 20 Å, which was centered on the centroid of Q203. MK9 was then docked using the Ligand Docking module. Finally, 10 docking poses were generated for MK9 after post-docking minimization based on the force field of OPLS3 (Harder et al., 2016). These poses were ranked by the scoring function of GlideScore. The pose with the lowest GlideScore was chosen for binding mode analysis.

### Binding free energies of Q203 between wild-type and mutant hybrid supercomplex

Binding free energies of Q203 between wild-type and mutant hybrid supercomplex were computed using the Molecular Mechanics/Poisson-Boltzmann Surface Area (MM/PBSA) method based on all-atom molecular dynamic simulations (Greene et al., 2019; Homeyer and Gohlke, 2012; Homeyer and Gohlke, 2015). This task can be divided into three phases: (i) simulation system construction, (ii) molecular dynamic simulation, and (iii) binding free energy computation.

Considering that QcrB is the core subunit interacting with Q203 in the hybrid supercomplex, the QcrB with Q203 bound was the only region extracted from the Q203-bound hybrid supercomplex for simulation system construction. Membrane Builder in CHARMM-GUI (Jo et al., 2007) was used to build the protein/membrane system solvated in water. PDB file containing the coordination information of Q203-bound QcrB was read while only protein residues and Q203 were retained. The CHARMM General Force Field (CGenFF) was applied to parameterize the Q203 (Vanommeslaeghe and MacKerell, 2012). DPPC lipid molecules were used to explicitly build the lipid bilayers and bulky water layers were added to the top and bottom sides of the membrane. In addition, 0.15 M KCl was added to neutralize the charge of system using the distance-based ion placing method. Next, the protein was inserted in the lipid bilayers and covered by the water layers despite exposure of some residues to the solvents. In order to generate topology and parameter files for subsequent AMBER dynamic simulations, the force fields of ff19SB (Tian et al., 2020) and Lipids17 (Lee et al., 2020a) were respectively applied to the parameterization of protein and lipid molecules. Four Q203-bound QcrB simulation systems were built, including wild-type QcrB, T313A mutant, E314Y mutant, and T313A/E314Y mutant.

Molecular dynamic simulation was carried out using AMBER 2020 (Case et al., 2005). Before dynamic simulation, 5000 steps of energy minimization were performed on waters to remove potential steric clashes between the solute and solvents. Harmonic restraints with a force constant of 10 kcal/mol Å^2^ and 2.5 kcal/mol Å^2^ were placed on protein and lipids in the minimization, respectively. Then, two stages of equilibrium simulations were carried out to relax the protein and lipid molecules by gradually decreasing the restraint force. The first stage was further divided into three short simulations each taking 375 ps with the time step set to 1 fs. The constant volume and temperature (NVT) ensemble was applied to this stage of simulation. The second one was a much longer simulation with the constant pressure and temperature (NPT) ensemble, which took 1.5 ns to completely relax the whole system with extremely low force. At this stage, the time step was increased to 2 fs while the SHAKE algorithm was used to fix the bonds with hydrogens (Bailey and Lowe, 2009). Subsequently, a 10 ns NPT simulation without any restraints was performed to further relax the system. Langevin dynamics was used to control the temperature at 303.15 K in NVT and NPT simulations (Grønbech-Jensen and Farago, 2014). In the NPT simulations, additional semi-isotropic pressure coupling and constant surface tension were applied to the simulation of lipid bilayers (Bennun et al., 2007). Once the equilibrium simulation was finished, a 100 ns NPT simulation free of restraints was carried out for each molecular system to produce the final trajectory used for energy calculation. Coordinates were printed every 100 ps so that in total 1000 frames were contained in the final trajectory. The CPPTRAJ module in AMBER was applied to analyze the trajectory (Roe and Cheatham, 2013).

MM/PBSA methods were employed to calculate the binding free energy of Q203 in wild-type and mutant QcrB (Homeyer and Gohlke, 2012). As shown in the equation (Δ*G*= *E*_MM_ + *E*_pol_ + *E*_np_ *- TS*), free energy can be calculated by combining the MM part (E_MM_) that represents the gas phase energy contribution as well as the solvation free energy components including polar (E_pol_) and non-polar (E_np_) contributions. The gas phase free energy was calculated according to the force field. As for the solvation free energy, the polar part representing electrostatic contribution was calculated using the Poisson–Boltzmann (PB) equation based on implicit solvent model (Honig and Nicholls, 1995). In AMBER, the equation also provides an implicit membrane model to calculate the membrane-mediated electrostatic interactions. Here, geometric multigrid based on iterative solver was selected for PB equation calculation (Harris et al., 2013). On the other hand, the LCPO method was applied to calculate the non-polar part that represents the hydrophobic contribution (Weiser et al., 1999). The Python script MMPBSA.py (Miller et al., 2012) in AMBER was used for the computation based on previously produced simulation trajectories, from which one frame was extracted every 1 ns for the energy calculations. Ions, water, and lipid molecules were all striped from the molecular systems before calculation. For implicit membrane model, the membrane dielectric constant was set to 7.0, and membrane thickness was set to 42 Å. Entropy contributions (TS) could be neglected as the four molecular systems were assumed to have similar entropy changes due to the same protein-ligand structures except for the mutated residues.

### Creation of figures

All the figures were created using UCSF Chimera (Pettersen et al., 2004) or PyMOL (Schrödinger LLC, 2017).

## Data Availability

All data generated or analysed during this study are included in the manuscript and supporting files. Source data files have been provided. The accession numbers for the 3D cryo-EM density map of apo, Q203-bound and TB47-bound hybrid supercomplex in present study are EMD-30943, EMD-30944 and EMD-30945, respectively. The accession numbers for the coordinates for the apo, Q203-bound and TB47-bound hybrid supercomplex in this study are PDB: 7E1V, PDB: 7E1W and PDB: 7E1X, respectively. The following dataset was generated: ZhouS WangW GaoY GongH RaoZ 2021Cryo-EM structure of apo hybrid respiratory supercomplex consisting of Mycobacterium tuberculosis complexIII and Mycobacterium smegmatis complexIVElectron Microscopy Data BankEMD-30943 ZhouS WangW GaoY GongH RaoZ 2021Cryo-EM structure of hybrid respiratory supercomplex consisting of Mycobacterium tuberculosis complexIII and Mycobacterium smegmatis complexIV in the presence of Q203Electron Microscopy Data BankEMD-30944 ZhouS WangW GaoY GongH RaoZ 2021Cryo-EM structure of hybrid respiratory supercomplex consisting of Mycobacterium tuberculosis complexIII and Mycobacterium smegmatis complexIV in presence of TB47Electron Microscopy Data BankEMD-30945 ZhouS WangW GaoY GongH RaoZ 2021Cryo-EM structure of apo hybrid respiratory supercomplex consisting of Mycobacterium tuberculosis complexIII and Mycobacterium smegmatis complexIVRCSB Protein Data Bank7E1V ZhouS WangW GaoY GongH RaoZ 2021Cryo-EM structure of hybrid respiratory supercomplex consisting of Mycobacterium tuberculosis complexIII and Mycobacterium smegmatis complexIV in the presence of Q203RCSB Protein Data Bank7E1W ZhouS WangW GaoY GongH RaoZ 2021Cryo-EM structure of hybrid respiratory supercomplex consisting of Mycobacterium tuberculosis complexIII and Mycobacterium smegmatis complexIV in presence of TB47RCSB Protein Data Bank7E1X

## Additional files

Transparent reporting form
