# Supplementary figures and images for: Structure of Mycobacterium tuberculosis cytochrome bcc in complex with Q203 and TB47, two anti-TB drug candidates
Source: eLife. 2021 Nov 25;10:e69418. doi: 10.7554/eLife.69418 (PMC8616580; doi:10.7554/eLife.69418)

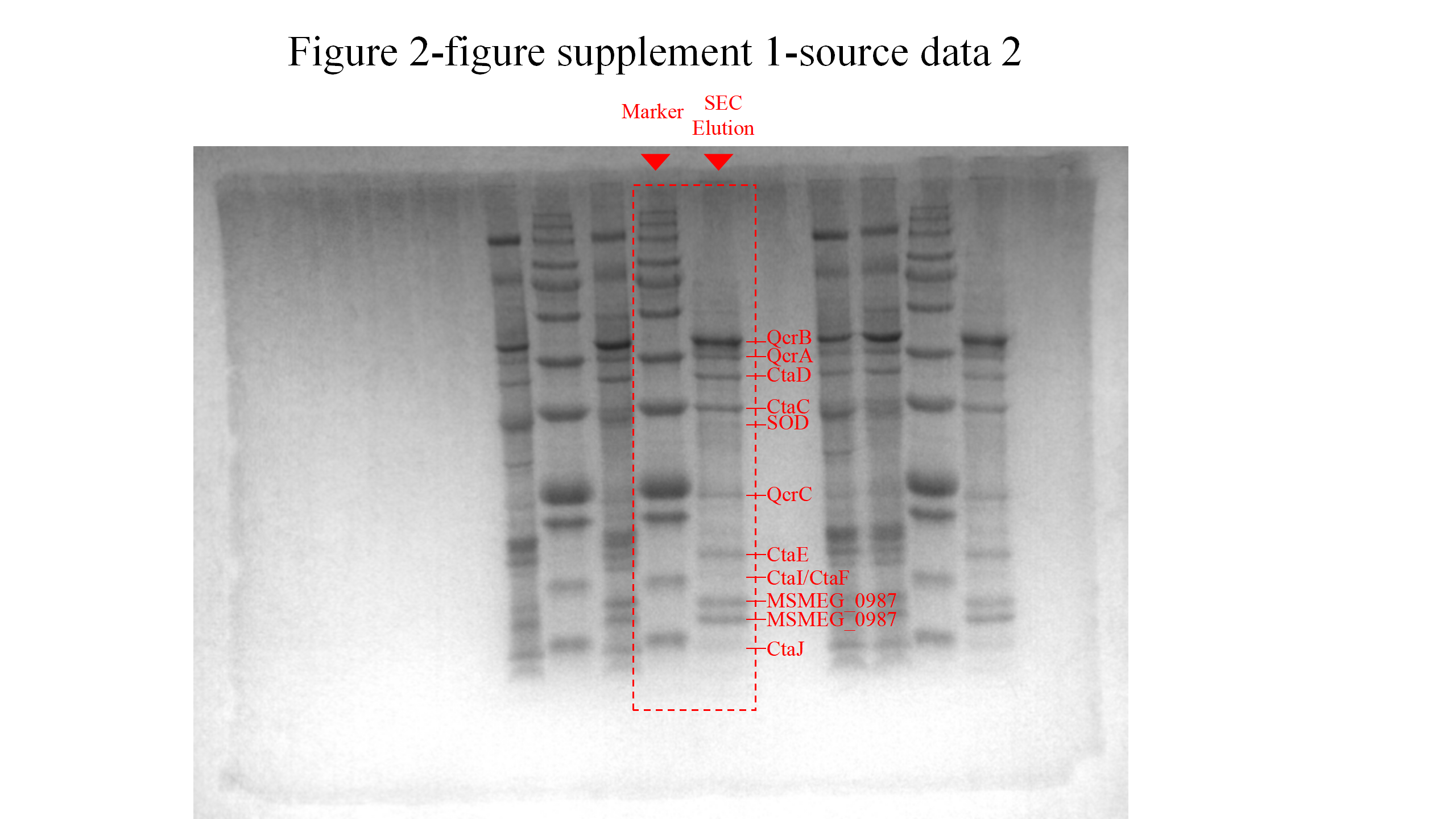

Supplement: Figure 2—figure supplement 1—source data 2. [file elife-69418-fig2-figsupp1-data2.tif]
